# Subaerial Decomposition of Small-Sized Remains in The Netherlands: Important Findings Regarding the PMI of a Four-Year Taphonomic Study

**DOI:** 10.3390/biology12091164

**Published:** 2023-08-24

**Authors:** Iris Sluis, Wilma Duijst, Tristan Krap

**Affiliations:** 1 Faculty of Law and Criminology, Maastricht University, Minderbroedersberg 4-6, 6211 LK Maastricht, The Netherlands; wilma.duijst@maastrichtuniversity.nl (W.D.); t.krap@maastrichtuniversity.nl (T.K.); 2 Forensic Laboratory Research, University of Applied Sciences Van Hall Larenstein, Agora 1, 8934 CJ Leeuwarden, The Netherlands; 3 GGD IJsselland, Zeven Alleetjes 1, 8011 CV Zwolle, The Netherlands

**Keywords:** forensic taphonomy, seasonal decomposition, small-sized piglets, post-mortem interval, subaerial decomposition, The Netherlands

## Abstract

**Simple Summary:**

Understanding changes that occur after death, or post-mortem changes, based on decomposition, is very important to relate them to a post-mortem interval (PMI), the time elapsed between the moment of death and discovery of a body. Most studies on decomposition focus on large cadavers, leaving a knowledge gap for small cadavers, which are representative for infants and subadults. To fill this knowledge gap, a season-based subaerial outdoor decomposition study was conducted with small pig cadavers at the Forensic Anthropological Outdoor Research Facility located in Den Ham, The Netherlands, over a period of 4 years. This study revealed important findings related to a deviating decomposition rate during winter and the subsequent spring, reproducibility, the effect of body weight, post-mortem movement, the effect of heavy rainfall on insect activity, delayed bloating, the interaction of different insect species, and invertebrate activity.

**Abstract:**

Studying post-mortem changes based on signs of decomposition (e.g., using scoring models) is one of the methods used in scientific studies to relate observable changes to the post-mortem interval (PMI). The majority of the studies on cadaver decomposition are based on large cadavers. There is limited literature on the decomposition pattern and rate of small cadavers, even though it is at least as important to be able to estimate the PMI for infants and subadults. Therefore, it is crucial to acquire knowledge of the decomposition process of child-sized remains. To fill this knowledge gap, a season-based subaerial outdoor decomposition study was conducted with small pig cadavers at the Forensic Anthropological Outdoor Research Facility located in Den Ham, The Netherlands, over a period of 4 years. Den Ham is located in the eastern part of the Netherlands, close to the German border, and has a temperate maritime climate, with a Cfb classification according to the Köppen–Geiger system. Salient findings were acquired during the decomposition study, specifically regarding a deviating decomposition rate during winter and the subsequent spring, reproducibility, the effect of body weight, post-mortem movement, the effect of heavy rainfall on insect activity, delayed bloating, the interaction of different insect species, and invertebrate activity. This article includes a systemic review of the results obtained during this four-year decomposition study and discusses the impact of the findings on the estimation of the PMI.

## 1. Introduction

Annually, on average, three deceased infants are found in the Netherlands, and this is considered to be only the tip of the iceberg according to the Netherlands Institute for the Documentation of Anonymous Abandonment (NIDAA) [1]. The finding of deceased infants is not only a problem in the Netherlands, but a worldwide issue [1,2,3]. When investigating deceased infants found in an outdoor context, several important issues must be considered, including viability, the presence of natural diseases or trauma, and the cause and manner of death. Answers to these questions are complicated by a process that starts from the moment an organism dies: decomposition [4]. However, the degree of decomposition can be used to estimate the post-mortem interval (PMI), the time elapsed between the moment of death and the moment of discovery of a body, to gather important information about the death and verify suspects’ statements in the tactical investigation. A reliable estimation of the PMI can contribute to the reconstruction of events leading to death (peri-mortem events) and the identification of an individual [5].

Decomposition consists of two processes, namely, autolysis, which is the breakdown of the body’s tissue by enzymes and internal chemical reactions characterized by an anaerobic environment (low oxygen); and putrefaction, which is the breakdown of body tissues by bacteria and fungi [6,7]. Decomposition can be divided into five different stages, namely: (1) the fresh stage, in which there are minimal signs of decomposition but discoloration can occur; (2) the bloating stage, involving gas formation; (3) active decomposition, where soft tissues decay (liquefaction); (4) the post-active stage, where decomposition progresses, mummification takes place, and dehydrated tissues (skin, cartilage, and bone) remain; and (5) skeletonization, in which bones are the end products [5,8,9,10,11]. Decomposition is an irreversible progressive process. However, several stages can be observed simultaneously within the same remains, for example, soft tissue decay takes place (stage 3), but mummification occurs in some places (stage 4), causing local decomposition to stagnate [12,13,14]. This can be explained by intrinsic and extrinsic factors that influence the decomposition of an individual. Intrinsic factors are variables that relate to the individual, such as the manner of death, trauma and wounds present, and body weight. Extrinsic factors are variables coming from outside the body that influence the decomposition process, such as humidity, oxygen level, ambient temperature, sunlight hours, precipitation, body cover, substrate, pH of the substrate, and insect activity [5,6,12,15].

In addition to insect activity, which has a significant impact on the rate and pattern of decomposition, temperature is one of the main factors influencing decomposition, as it is the primary factor for all biological activities and biochemical reactions [5,6,12,16,17]. Temperature can also have an effect on the bloating of the body. The bloated stage involves the build-up of gases in the body, resulting in putrefaction. The bloated stage is considered the second stage of decomposition and usually begins 48 h after death and lasts on average around seven days, with an extremity of 13 days, partly depending on the ambient temperature [10,12,18,19,20]. In addition to ambient temperature, the size of the cadaver plays a role in the inflation. The bloated stage of larger cadavers may start earlier and last longer, which is probably related to larger and more complex microbials and slower heat loss [19]. Low temperatures reduce or stop decomposition and can also inhibit insect activity and bacterial growth [6,12,17,21,22]. Therefore, decomposition progresses slower in the colder months than in the warmer months [23]. Freeze and thaw cycles are taphonomic processes that directly influence decomposition [24]. Cycles of freezing and thawing affect the decomposing tissue and bacteria present in the cadaver, causing the body to go through stages of inflation and deflation. After the corpse has thawed, decomposition can quickly recur under the right conditions but has a different rate of tissue liquefaction compared to a body with a generally equivalent decomposition time that has not been exposed to a temperature below zero [17,25,26]. Humidity and rain also play an important role in the decomposition process. A higher atmospheric humidity is favorable for both decomposition and maggot activity, and the chance of mummification is reduced [16,20,27]. Considering the effect of rain on decomposition, there is no uniform outcome from the literature. Some studies state that rain affects insect activity and oviposition and, therefore, decomposition because maggots might be expelled from the wet carcass [20,28,29]. Other studies argue that rain does not affect insect activity, because most maggots retreat into body cavities and are able to continue feeding in this way while remaining protected from temperature changes, wind, rain, snow, and solar radiation [12,16,30]. Blowflies are attracted to a body within the first hour after death, but rain is a clear deterrent to laying eggs [31,32]. There is also no uniform outcome regarding the exact effect of weight on decomposition, even though several studies have investigated this effect [21]. Some studies suggest that body weight would have no direct effect on how decomposition will proceed because the fats present in individuals with higher body weight (110 kg versus 65 kg) liquefy during decomposition [16,33]. Studies stating that individuals with a larger body weight decompose faster suggest that there is a higher amount of fluid in the tissues in larger individuals, which promotes the development and spread of bacteria [12]. In addition, subcutaneous fat and abdominal fat have insulating properties and therefore slow down the cooling rate of the body; higher body temperatures can result in accelerated decomposition and a thinner person cools more quickly because of both the mass-to-surface ratio and the lack of fatty insulation [13,23,34,35]. On the contrary, other researchers argue that smaller bodies decompose faster, which would be due to insects having to do more ‘work’ on larger bodies [21,36].

Research by Gelderman et al. (2021) has shown that PMI estimations based on decomposition of remains of adults are unreliable [37]. It is expected the same holds for remains of subadults. In addition, scoring models used to estimate the PMI are based on and intended for larger cadavers and there are no models for the decomposition of smaller cadavers. The use of decomposition stages is one of the methods that can be used to estimate the post-mortem interval. Galloway et al. laid the basis for assessing decomposition. That basis was subsequently converted into a scoring model by Megyesi et al., who also introduced a formula to estimate the post-mortem interval from the decomposition score for adult bodies that decomposed subaerially. Decomposition is scored for three body regions (head and neck, trunk, extremities), and the sum of these scores is called the Total Body Score (TBS) [10,11]. The scoring model of Megyesi et al. was developed for the climate of the United States, and it was stated that future research should focus on other parts of the world to map environmental and climate conditions related to the scoring model, as the use of seasonal results from other regions can lead to inaccurate PMI estimates [6,11,22,38,39]. The model was previously tested for other countries outside Europe (Canada and Australia, all for large cadavers) [40,41,42].

The research aim of this study was to investigate the outdoor seasonal decomposition of small cadavers in the Netherlands. Therefore, we used the scoring model of Megyesi et al. for the Netherlands and on small cadavers, as this has not been done before in the Netherlands or neighboring countries [11]. The main findings of the seasonal decomposition study were subjected to a further structured-finding-based systematic literature search, in which the obtained literature was subsequently assessed and reviewed.

## 2. Materials and Methods

**Figure 1:**
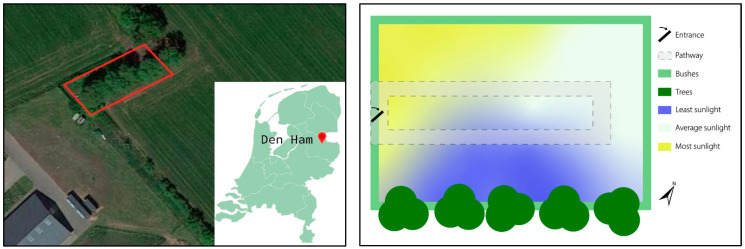
The Forensic Anthropological Outdoor Research Facility Den Ham. The left figure shows where the research location is situated. The figure on the right shows the average sunlight distribution at the research location. The pathway is also shown, around which the cages with pig cadavers were placed.

In 2015, a research location (an outdoor laboratory) in Den Ham (province of Overijssel), called the Forensic Anthropological Outdoor Research Facility (FAORF-DH), was set up for decomposition studies; see Figure 1. Permits were provided by the Dutch Food and Safety Authority (NVWA), the Water Authority, and the municipality. The FAORF-DH was established through a collaboration between the University of Applied Sciences Van Hall Larenstein Leeuwarden, Ars Cogniscendi for forensic and legal medicine, and the Veterinary Knowledge Centre East Netherlands. From the beginning of summer 2015, a subaerial (situated on or immediately adjacent to the surface of the earth) decomposition study was started at the FAORF-DH, which lasted until the spring of 2019.

Domesticated pig (*Sus scrofa dom.*) cadavers were used as a substitute for humans because of their physiological and anatomical similarity to humans [43,44,45]. The population consisted of deceased pigs (piglets died as a result of the mother pig lying on the piglet, smothering them/causing internal trauma) and stillborn pigs, all from a biological farm; hence, no antibiotics or other medications were administered during life. The remains were donated to the study for compensation of expenses (consisting of storage and transportation to the site). The pig cadavers were divided into two different weight classes. The first weight class (hereafter category 1) ranged within 800–2700 g and the second weight class (hereafter category 2) ranged within 4230–10,500 g. Category 1 represents the weight of neonatal remains, and category 2 represents the weight of remains up to 3 years of age, based on the torso [43]. Different weight categories were also chosen to be able to investigate the influence of weight on the decomposition pattern and rate. The present study defines the decomposition pattern as the progression of a cadaver through the different decomposition phases. The manner in which the decomposition score differs per anatomical region compared to another anatomical region of the same cadaver, and compared to other cadavers, was examined. The decomposition rate is defined as the increase in the Total Body Score (TBS) relative to the time. The decomposition rate is expressed as the slope of the trendline of the season-based results, where a higher slope means that the Total Body Score reaches a higher value in fewer days and thus has a higher decomposition rate. Four pig cadavers were placed on location at the beginning of each season, depending on the day the pig died. A total of 64 pig cadavers were placed during all four years of the research, namely, four piglets per season, of which two pig cadavers were from each weight category (*n* = 64; per weight category *n* = 32). Shortly after the pigs had died, they were stored in a refrigerator (4 degrees Celsius) for a maximum of one day and were then placed on location. To be included in the current research, the pig cadavers had to align with the weight categories mentioned earlier. Furthermore, they were required to show no signs of decomposition, and a veterinary examination was conducted to exclude any indications of infections in the piglets and any external injuries. The cadavers were placed naked and uncovered on-site and were left to decompose under natural environmental conditions. For this purpose, the cadavers were placed in separate wire mesh cages (2.5 m apart from fresh to post-active decay) to protect against vertebrates (predators) without a barrier to insects and were equipped with a thermometer. Decomposition was scored on location using the Megyesi et al. scoring method [11] (see Table 1) and the remains were photographically documented multiple days a week. In addition, external variables were also documented, namely, temperature (on-site and at a weather station at Heino, Overijssel, The Netherlands), precipitation, hours of sunshine and shade, soil pH, and insect activity. The external variables that were applicable in the present study can be listed from most influential to least influential and were temperature, access by insects, humidity, rainfall, and body size and weight [16]. Den Ham is located in the eastern part of the Netherlands, close to the German border, and has a temperate maritime climate, with a Cfb classification according to the Köppen–Geiger climate classification system. The average annual temperature is 10.5 °C and there is almost 1000 mm (840 mm) of rainfall in a year, with a possibility of rainfall during all months of the year. There is an average humidity of 82% and a UV index of 3. The map on the right in Figure 1 shows the average sunlight distribution at the research location. For this research period, the average minimum and maximum temperatures (in degree Celsius) for the summer season were, respectively, 11.80 (SD = 2.69) and 22.65 (SD = 3.92); for the autumn season, 5.14 (SD = 4.37) and 12.86 (SD = 5.90); for the winter season, 3.66 (SD = 5.31) and 13.01 (SD = 6.94); and for the spring season, 6.37 (SD = 4.80) and 17.40 (SD = 5.51).

In addition to the on-site temperature data, weather data (including precipitation) from the local weather station (Heino, Overijssel) were collected, obtained through the Royal Netherlands Meteorological Institute (KNMI) [46]. In this way, it became possible to compare the on-site data with the KNMI data, in order to explain or support certain findings (for example, to determine if any anomalous temperature data in specific years could explain deviating findings). Data from the decomposition study were visualized using the SPSS version 27 software. The data were processed into graphs and scatterplots, in which the time was plotted against the average TBS, to map the decomposition rate and pattern per season and to determine the reproducibility of the same seasons in consecutive years. Further, linear regression was performed to establish the correlation, slope, and Residual Sum of Squares (RSS). The correlation scores are between 0 and 1, and can be divided as follows: 0.00–0.40, 0.41–0.59, 0.60–0.74, and 0.75–1.0, which indicate a poor, fair, good, and excellent correlation, respectively [47]. The Residual Sum of Squares (RSS) is a measure of the level of variation in the error term of the regression model. The lower the RSS, the better the models fit the data [48]. Some cadavers reached a plateau phase in the TBS after a given period (days). A plateau phase was seen as a sequence of a minimum of four data points with the same TBS in the advanced decay phase, without increasing afterwards. A slope cut-off was therefore applied upon reaching this plateau phase in the advanced decay, defined as a period (days) of stagnation of the TBS. The plateau phase did not arise with every cadaver at the same TBS as not all cadavers reached the maximum TBS of 35. In addition, the winter cadavers never reached a plateau phase. In order to indicate the TBS values of the different cadavers in the seasons, the average maximum TBS reached was calculated for the different research years. The output was interpreted, along with photographic documentation, to map out the key findings from this decomposition study. These findings were subsequently subjected to a systematic literature review to determine the similarities and discrepancies with decomposition studies in other contexts (see Supplementary Materials Table S1 for the search details).

## 3. Results and Discussion

### 3.1. Decomposition Results and Interpretation

In this chapter, the results of the current study are presented, divided into seven interesting findings. These findings were compared to the current international literature. The comparison with the current international literature is presented in the Supplementary Materials Section S2.

#### 3.1.1. Decomposition Rate

**Figure 2:**
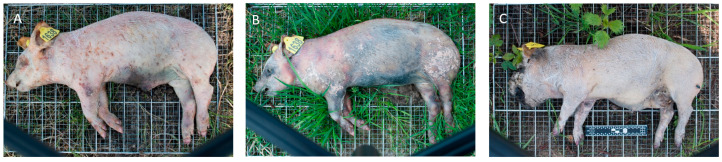
Visual appearance of pig cadavers: (**A**) pig cadaver, winter season 38 days post-mortem, which is in the stage of active decomposition; (**B**) pig cadaver, winter season 92 days post-mortem, which is in the post-active stage; (**C**) pig cadaver, spring season 38 days post-mortem, which is in the post-active stage.

**Figure 3:**
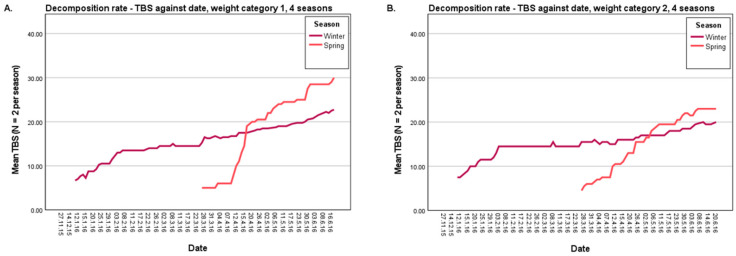
Mean Total Body Score (TBS) for the winter and spring seasons versus time (*n* = 2 per season). (**A**): Research years 2015–2016 weight category 1 (800–2700 g). (**B**): Research years 2015–2016 weight category 2 (4230–10,500 g).

The results of the four-year decomposition data show that, based on the average TBS of four cadavers per season, differences in decomposition rate can be noted. In general, the decomposition rate of winter cadavers was lower than in the other seasons. In addition, it can be noted in almost all years that there was an overlap in mid-April between the decomposition score of the winter cadavers and that of the spring cadavers. The winter cadaver was in the active decomposition stage after 38 days post-mortem (Figure 2A) and in the post-active stage after 92 days post-mortem (Figure 2B). The cadaver of the spring season was also in the post-active stage after 38 days post-mortem (Figure 2C) and still in the post-active stage after 92 days post-mortem. From the moment of the overlap, it was therefore no longer possible to distinguish between a winter cadaver and a spring cadaver based on the scoring model of Megyesi et al., while the winter cadaver had been decomposing for 54 days longer compared to the spring cadaver. In Figure 3, the average TBS is plotted against time, and both the winter and spring seasons are shown (Figure 3; 2015/2016, see Supplementary Materials Figure S1 for the other years and all seasons). Here, the difference in decomposition rate can be clearly observed between the winter and spring seasons, where from mid-April an overlap is visible. However, there was an exception for the larger (weight category 2) winter and spring cadavers of 2017, where the lines did not cross (see Supplementary Materials Figure S1D). There was another exception of the larger (weight category 2) autumn cadavers of 2017, where the autumn period started to overlap with the winter and spring periods of 2018 (see Supplementary Materials Figure S1F). Based on these overlapping results, it would not be possible to distinguish an autumn/winter/spring cadaver for those periods, and thus the post-mortem interval estimation based on the Total Body Score would not correspond to the actual PMI.

#### 3.1.2. Reproducibility

**Figure 4:**
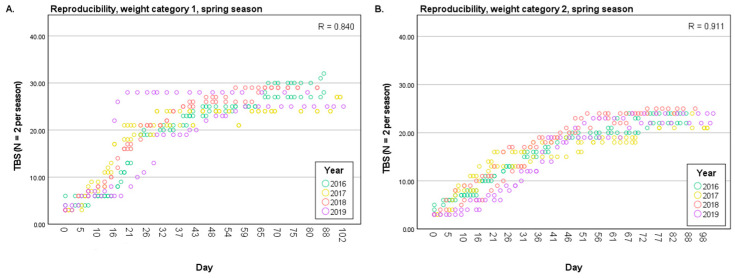
Total Body Score of the pig cadavers against the number of days post-mortem, where all research years are shown (*n* = 2 per season), to show the progress in decomposition. (**A**): Spring season weight category 1. (**B**): Spring season weight category 2.

The results regarding the decomposition pattern and rate of this decomposition study (2015–2019) are generally comparable. Further, the results show high correlations, little spread, and the same course of decomposition (see Figure 4; spring season and Supplementary Materials Figure S2; other seasons). There was a good correlation for the summer season, an excellent correlation for the smaller autumn cadavers, the winter season, and the spring season, and the correlation for the larger autumn cadavers was fair (see Table 2 and Supplementary Materials Table S2). Little variation and spread in the models were also confirmed by the Residual Sum of Squares (RSS). Most scattering was found for the larger autumn cadavers, and the least spread was found for the larger spring cadavers (see Table 2 and Supplementary Materials Table S2), corresponding with the correlation coefficients.

#### 3.1.3. Body Weight

The pig cadavers used in this study were divided into two body weight categories of 800–2700 g and 4230–10,500 g. Based on the average progression of the PMI in days through all decomposition phases, the decomposition rate of the smaller cadavers was generally higher in the autumn, winter, and spring seasons than that of the larger cadavers, and the decomposition rate of the larger cadavers was higher during the summer, but there was a slight difference compared with the smaller cadavers (see Figure 4; spring season and Supplementary Materials Figure S2; other seasons). To further investigate this observation, the slopes of the graphs were determined (see Supplementary Materials Table S2). As explained in the methodology, it was decided to implement a slope cut-off as soon as a cadaver reached a plateau phase in the advanced decay. This resulted in data including the plateau phase (see Supplementary Materials Table S2) and data excluding the plateau phase (see Supplementary Materials Table S3). Not all cadavers reached a plateau phase, so the TBS was not cut off. By cutting the TBS in the plateau phase, a higher slope was obtained; see Table 3 for all research years together and Supplementary Material Tables S2 and S3 for all individual research years. In addition, it was found that the smaller cadavers generally reached the active stage earlier in the summer, autumn, and spring periods. Decomposition during winter followed a similar pattern for the smaller and larger cadavers. Based on these results, it appears that there is a difference in decomposition rate and pattern between the larger and smaller cadavers and that this also differs between the seasons. Additionally, the applicability of an estimation method depends on whether or not a plateau is reached. Hence, the rate of decomposition depends on the intrinsic variable weight and the external variable temperature.

#### 3.1.4. Post-Mortem Movement

**Figure 5:**
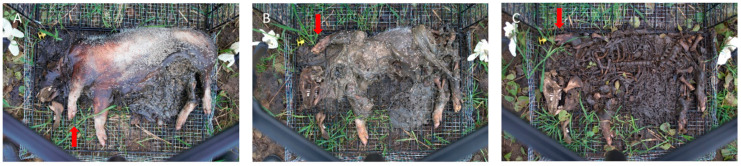
Post-mortem movement observed in a pig cadaver in the autumn season, as indicated by the red arrows. (**A**) Position of cadaver the same as when placed, 21 days post-mortem. (**B**) Left forelimb has migrated overhead, 28 days post-mortem. (**C**) Leg remains in this position until the final stage of decomposition is reached, 36 days post-mortem.

Post-mortem ‘movement’, i.e., displacement of body parts relative to each other, was observed in a cadaver of the autumn season of 2015. After 28 days post-mortem, it could be observed that the left forelimb, which was inferior to the head when the cadavers were placed (Figure 5A), has moved to a position superior to the head (Figure 5B). From then on, the leg remained there, as shown in Figure 5C. The explanation for this was found in exposure to sunlight, resulting in a locally higher temperature on the dorsal side resulting in the mummification of soft tissues. Mummification is caused by dehydration, and tissues that dehydrate decrease in volume. However, active decomposition continued to take place on the ventral side. Insects often migrate to shaded areas, because of lasting moisture, and, in this way, they are more protected/less noticeable to predators [49]. Hence, dorsal shrinkage and loss of ventral soft tissue integrity resulted in tensile forces without resistance, resulting in the displacement of the right forelimb.

#### 3.1.5. Rainfall

**Figure 6:**
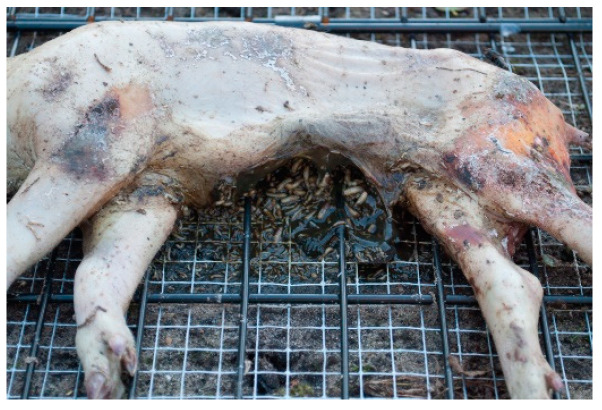
Heavy rainfall affected the maggot activity of an autumn cadaver of 2015, resulting in stopped maggot activity and a delay in decomposition rate.

During the autumn season of 2015, there were periods of rainfall, which mainly affected the small cadavers. A maggot mass that was present on a pig cadaver from the autumn season turned out to be inactive after this period of rainfall; a large part had died, and another part had possibly migrated away from the cadaver; see Figure 6. This can also be seen in Figure S1A of the Supplementary Materials; autumn season 2015 (green line), where a plateau phase arose between 14 October 2015 and 4 November 2015. Insect activity is a strong influencing factor for the rate of decomposition [16]. In this study, prior to the plateau phase, there was active insect activity, without precipitation. The start of the plateau phase was accompanied by the arrival of rainfall and the insect mass began to die. The plateau phase then stopped when a new entomological phase started. By comparing the rainfall data from the KNMI and the on-site data, it was established that, from the start of the plateau phase, 0.5–9 mm of rain fell, with a local storm generating a peak in precipitation of 15 mm (on 10 October 2015, measured on-site only) [46]. As a result, it could be stated that moderate rainfall, with a local storm generating a peak precipitation of 15 mm, affected the maggot activity (i.e., it stopped this activity) and delayed the decomposition rate.

#### 3.1.6. Delayed Bloating

Delayed bloating, i.e., a delay in the start of the bloating phase (delayed start compared to other seasons), and an extended bloating period (bloating phase lasted longer), was observed in the smaller cadavers (category 1) in the winter and spring seasons and in the larger cadavers (category 2) in the autumn, winter, and spring seasons. For example, for a cadaver (category 2) that was placed in the winter season, the bloating stage was not reached until 88 days post-mortem and persisted until 106 days post-mortem. Here the cadavers of the spring season in the chronology would have already passed this stage, while this stage of the winter cadavers had not yet started. Table 4 shows an overview of the number of days the bloating stage lasted on average per season over a period of four years. It could be argued here that the ambient temperature has a significant influence on this stage of decomposition, both on the period in which it starts and on the length of time it lasts.

#### 3.1.7. Invertebrate Activity

**Figure 7:**
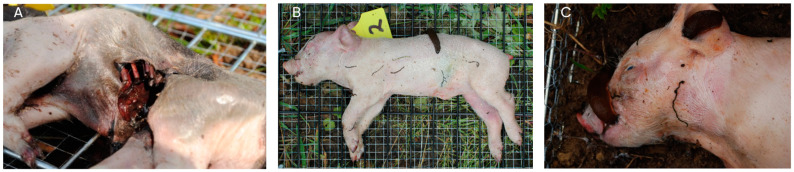
(**A**) An example of the skin lesions caused by the wasps, in which the flies laid eggs. Here you can see a cadaver of the spring of 2015 (category 1), four days post-mortem. (**B**) An example of the slugs that were present on the cadavers. Here you can see a cadaver of the summer of 2015 (category 1), two days post-mortem. (**C**) An example of the slugs that were present on the cadavers. Here you can see a cadaver of the summer of 2018 (category 1), one day post-mortem.

Interactions between different invertebrates were observed. The invertebrates present at the decomposition were slugs, wasps (Hymenoptera), three cycles of maggot succession (Diptera), and beetle larvae (Coleoptera), which were highly active. The interaction between wasps and flies was remarkable. The wasps caused skin lesions and coarser injuries to the tissue, which ultimately allowed the flies and their offspring easier access (see Figure 7A). In addition, wasps prey on the maggots. The slugs, wasps, maggots, and beetles were present in each season, where the slugs were observed at a total of nine cadavers over the entire study period. For four cadavers, these were the first days post-mortem (day 0–4), and for the other four cadavers, this was later in the decomposition process (11, 18, 19, and 42 days post-mortem). The slugs were mainly interested in the softer parts of the cadaver and were mostly found on the abdomen, groin, ears, nose, and the rest of the face (see Figure 7B,C).

### 3.2. Discussion

This article discusses the findings made during a four-year seasonal decomposition study using pig cadavers in the Dutch climate. The research aim of this study was to investigate the outdoor seasonal decomposition of small cadavers in the Netherlands. Therefore, we tested the scoring model of Megyesi et al. for the Netherlands and on small cadavers, as this has not been done before in the Netherlands or neighboring countries [11]. Several outcomes were obtained during this study related to the deviating decomposition rate during the winter and spring period, the reproducibility of the results, the effect of body weight, post-mortem movement, rainfall that affected insect activity, delayed bloating, the interaction of different insect species, and invertebrate activity. These factors influenced both the decomposition pattern and rate of small pig cadavers in the Dutch climate. This makes it interesting to reflect whether these findings are also observed in other contexts or are specific to small cadavers or the Western European mainland. The Supplementary Materials contains a systematic review of the various subtopics. The discussion below is based on this review.

#### 3.2.1. General Discussion

A lower decomposition rate during the winter season resulted in a slower progression of decomposition compared to the subsequent spring season. As a result, the winter season started to overlap with the spring season from mid-April onwards. This was not observed in other studies, making this finding relevant for practice. This finding may therefore be specific to small cadavers or the Western European mainland. This phenomenon indicates that climate- and season-related differences influence the rate of decomposition to a great extent and, thus, the estimated PMI. It particularly affects the accuracy and precision of estimating the PMI in the period from the onset of winter. If the post-mortem interval was estimated based on this data, it would be very large and imprecise for the winter period and very inaccurate (too precise and therefore inaccurate) for the spring period. The decomposition rates during summer and autumn seasons were generally comparable, which was also seen in the research by Giles et al. 2020 [27]. However, there were two exceptions to this finding, both for 2017: one where winter and spring did not overlap, and the other where autumn also started to overlap with winter and spring. We examined whether different climatic conditions, such as temperature, could have had an influence on the exceptions to the finding, but no exceptional explanations were found for the winter, spring, and autumn of 2017 that differed from the other years. It is striking, however, that both exceptions were only for the larger cadavers (weight category 2) and the results of the smaller cadavers were in line with those of the other years.

The results of the four-year decomposition study were generally comparable and high correlations could be observed. A combination of inter-observer variation and small climate-related differences can explain the variation between the different years of the same season, which may explain the degree of correlation. However, this does not explain the variation between the different weight categories, since decomposition was observed by the same researchers and the climatic conditions were similar. It is therefore striking that there was a difference in the degree of correlation between the size of the cadavers and the different seasons, but that there was generally a moderate to excellent correlation. Decomposition studies with pig cadavers generally have a higher reproducibility than those using human donors because the variation between bodies of pigs is smaller than that in humans. The pigs used in the studies often come from one breeder, have the same diet and intestinal flora, have a similar pattern of exposure to certain pathogens, and are equal in body weight [50,51]. In addition, the genetic diversity is smaller and the heterozygosity is relatively lower in pigs than in humans [52]. While the variation in babies and children is still relatively small, this is already much greater in young adults and adults, as the variation increases with age. This is partly due to BMI, lifestyle, diet, and underlying variables. It is therefore to be expected that a taphonomic model developed for babies and children has a smaller spread in data than a model for (young) adults. The degree of influence of the intrinsic variables on the decomposition rate decreases relative to the extrinsic variables. If a taphonomic model was developed for babies and children based on pigs, this model would be more accurate and precise than a model for (young) adults. However, this model still needs to be developed and subsequently validated.

Based on the average progression of the PMI (in days) through all decomposition phases, the decomposition rate is dependent on the intrinsic variable weight and the external variable temperature. In the literature, there is no uniformity about whether the decomposition rate of smaller or larger cadavers is higher [19,53,54,55,56]. In this study, therefore, the season in which decomposition took place was also taken into account to examine whether certain results emerged concerning the weight of the cadavers. No studies were found in this literature review that included this, meaning that this finding is specific to the Western European mainland or not sufficiently researched in other contexts.

The post-mortem movement observed in autumn cadavers was caused by the mummification of tissue. The conclusion made by Wilson and her colleagues in Australia, where the explanation of post-mortem movement was also found to be related to the mummification of tissues, is in line with the finding in the Netherlands and is therefore also applicable in the Dutch context [57]. The difference, however, is that the study by Wilson et al. used human adults and the present study used small-sized pig cadavers.

In this study it was found that moderate rainfall, with a local storm generating a peak precipitation of 15 mm, affected the maggot activity (i.e., it stopped this activity) and delayed the decomposition rate. Initially, it was thought that heavy rainfall did not influence the entomological activity [12,16,30]. However, there are also studies stating that rain affects insect activity and oviposition because blowflies and maggots are expelled from the wet carcass [20,28,29]. Our study obtained a similar result to that of the study conducted by Lyu et al. 2016 in China with *Sus scrofa* L., but using a different pig species than we used, which also showed a delayed decomposition rate in smaller cadavers [28]. This finding may therefore be specific to small cadavers and is important because it appears that rainfall can affect the entomological activity and thus the decomposition rate.

Delayed bloating was observed in the smaller cadavers (category 1) in the winter and spring seasons and in the larger cadavers (category 2) in the autumn, winter, and spring seasons. Both the study by Matuszewski et al. 2010 and the study by Díaz-Aranda et al. 2018 observed that the bloating stage in the colder months (winter and spring periods) started later and lasted longer [18,58]. Consequently, the advanced decay started much later and continued into the following season; this is consistent with the finding made in the current study, which also observed delayed bloating in the winter and spring seasons. However, the studies by Matuszewski et al. 2010 and Díaz-Aranda et al. 2018 both used large cadavers (14+ kg), while the current study used small cadavers with a maximum weight of 10.5 kg [18,58]. A finding not documented in the existing literature is that delayed bloating in this study was also observed in the autumn period for the cadavers in weight category 2 (4230–10,500 g), which may therefore be specific to the Western European mainland or small cadavers. The delayed bloating of the cadavers in, for example, the winter season, means that cadavers from the spring season in the chronology would already have passed this stage, resulting in an inaccurate time since death, when a winter cadaver is mistaken for a spring cadaver.

The observed interaction between the wasps (Hymenoptera), which modify and open the cadaver tissue, where the flies then lay eggs, and the wasps prey on the maggots, is consistent with the category of omnivores that includes wasps. This finding is therefore consistent with what is seen in the existing literature and forensic entomology. The slug activity observed in this study was primarily in the fresh stage, where the slugs were mainly present on the face, abdomen, and groin. This is in line with the slug activity observed in the study by Šuláková and Barták 2013 [59]. However, the forensic relevance of this slug activity is still unclear due to the lack of correlation with the specific phase of decomposition [59] and should therefore be further investigated.

#### 3.2.2. Limitations of the Study

There is now an outdoor decomposition research facility in the Netherlands for subaerial decomposition studies. This facility this promotes forensic taphonomical research in Western Europe. However, the outdoor decomposition research facility has a limited available area, where a maximum sample size of *n* = 4 per season (two cadavers per weight category) could be placed to maintain sufficient distance between the pig cadavers [50]. In this study, linear regression was used to analyze the results. A larger sample size would increase the reliability of the results, but maintaining sufficient distance between the cadavers would not have been feasible in the current study [60]. In addition, some of the findings within this study were limited to a single cadaver, namely the post-mortem movement and the rainfall that affected maggot activity. Since decomposition studies involve variability in decomposition, these are important findings within a bigger sample size since they could also occur in practice [55,60].

Both stillborn and deceased pig cadavers were used in the study. The deceased pigs died because of smothering/internal trauma caused by the mother, as the mother pig had been laying against the piglet. There may be a difference between these two categories, as the lungs of a stillborn piglet may not be fully developed and the bacteria in the digestive tract may be different between the two [4]. Since in real-life situations there may also be stillborn babies or babies that died after birth, it was decided to continue to use both categories in this study. The use of pigs as a proxy for babies and children was a considered choice because it is not allowed nor possible to use babies or children for decomposition studies. Of course, piglet models cannot be fully extended to human taphonomy, but can be used as a substitute because of their physiological and anatomical similarity to humans [43,44,45]. All piglets were medically approved by a veterinarian before the start of the study and therefore all have a health certificate. This suggests, for example, that there was no indication of an infection and that the piglets did not show any external injuries.

There are also some notable points about the scoring model of Megyesi et al. For example, the model was developed for human decomposition, and the study by Marhoff et al. 2016 found that the scoring model was not accurate for assessing the decomposition in pigs. It was found that the PMI was underestimated for all samples tested, as the published equations proved to be ineffective [40]. However, this does not affect the current study because only the decomposition was scored using the scoring model and no PMI was calculated in the present study. Additionally, we did not consider using the revised scoring model by Keough et al. 2017, which was specifically developed for pig models, since the current study started in 2015 and the scoring model of Keough et al. was published in 2017 [51]. By that time, the current study had already been progressing for at least two years, and we thus decided to proceed with the scoring model of Megyesi et al. An interesting future step would be to retrospectively apply the scoring model of Keough et al. using the available photographic material to determine if this model performs more accurately than the scoring model of Megyesi et al., which was developed for human adults. The same applies to the scoring model of Gelderman et al., which is used to score the decomposition of adults in the Dutch climate, and was developed in 2018. With this scoring model, the first step has been taken in developing a model that can estimate the PMI for adult decomposition in the Dutch Climate [5]. With the current research, we want to expand the knowledge about the decomposition pattern and rate for infants and subadults in the Dutch climate. It would thus be interesting to retrospectively apply the scoring model of Gelderman et al. to photographic material in order to investigate the accuracy and precision of the model for infants and subadults.

#### 3.2.3. Further Research

For this study, the outdoor seasonal decomposition of small cadavers in the Netherlands was investigated. The results of this first decomposition study show that there is high reproducibility, resulting in high precision. This was also expected as pig studies have higher reproducibility and less variation than human decomposition studies [50,51]. As there is no consistent outcome in the literature regarding the effect of weight and size on the progression of decomposition, it is important to conduct decomposition research with small-sized remains in addition to studies involving adults. It is believed that the decomposition rate of infants is nearly twice as high as that of adults. These differences are attributed to overall size, body mass, and surface-to-volume ratio [43,54,55]. While infants are smaller compared to adults, they generally have a relatively higher proportion of adipose tissue (body fat) compared to adults. The amount of fat in human infants is comparable to that in small piglets. Human infants have a fat percentage of 15%, and four weeks after birth, small piglets have an equivalent amount of fat [61,62,63]. This parallel between infants and small piglets may therefore be relevant in decomposition studies, as this can lead to variations in decomposition pattern and rate compared to adult decomposition.

For follow-up research, we want to correlate the Total Body Score with accumulated degree days (ADDs) to predict a post-mortem interval. The ADD is calculated by adding up the average daily ambient temperatures from the day of death to the day the body is discovered. It serves as a quantification of the thermal energy required for the biological and chemical processes involved in the decomposition of a body. By considering the ADD, a PMI can also be estimated, which is expected to be more accurate as temperature is believed to be the most important factor; however, it also requires an additional step, namely, calculating the ADD from the date of discovery back until the estimated date of death [5,36]. Once the baseline regarding decomposition pattern and rate has been created, the first step can be taken to develop a model that can estimate the post-mortem interval, and then to investigate how accurately this model works. For this, it is necessary to investigate the internal and external variables included in this study that influence the decomposition pattern and rate, and thus the precision of the PMI model. As a next step, certain variables (such as clothing) could be added that were not included in the current study, and the effect of these variables on the decomposition pattern and rate could be investigated.

The results of this study show that there are findings regarding the decomposition pattern and rate of small cadavers that have not been mentioned in publications relating to other contexts. These findings influence the post-mortem interval estimation and should therefore be taken into account when the PMI is estimated. The study, therefore, provides new and relevant knowledge and insights that can and should be applied in primary forensic practice. The results are thus important for casework and should be taken into account when dealing with decomposing remains and estimating the post-mortem interval. To avoid under- and overestimation of the estimated post-mortem interval and to obtain more knowledge about the decomposition pattern and rate of small cadavers, it is important to carry out follow-up research.

## 4. Conclusions

The results of this subaerial outdoor decomposition study show that there are findings that might be specific to the Netherlands, and possibly extending to a larger part of the Western European mainland, as well as for small cadavers regarding the decomposition pattern and rate.

The degree of reproducibility, post-mortem movement, delayed bloating in the winter and spring seasons, interaction between different insect species, and invertebrate activity corresponded to previous results on decomposition in other contexts worldwide.

To the best of our knowledge, the deviating decomposition rate during winter and the subsequent spring, and the effect of body weight, have not been recorded or mentioned in other studies. Further, the effect of heavy rainfall on insect activity and the delayed bloating in the autumn season may be specific to small cadavers.

In order to gain more insight into the accuracy and precision of the scoring models, such as those of Megyesi et al. (2005) and Gelderman et al. (2018), and therefore their applicability for daily casework, it is important to start follow-up research. Further, post-mortem movement and the effect of heavy rainfall on insect activity are currently understudied. In this way, the demonstrable new relevant knowledge and insights could and should ultimately be applied in primary forensic practice and, subsequently, in the judiciary.

## Figures and Tables

**Table 1:** Categories and stages of decomposition from Megyesi et al., 2005 [11].

**Categories and Stages of Decomposition for the Head and Neck**
A. Fresh - (1 pt) Fresh, no discolortion
B. Early decomposition - (2 pts) Pink-white appearance with skin slippage and some hair loss. - (3 pts) Gray to green discoloration: some flesh still relatively fresh. - (4 pts) Discoloration and/or brownish shades particularly at edges, drying of nose, ears and lips. - (5 pts) Purging of decompositional fluids out of eyes, ears, nose, mouth, some bloating of neck and face may be present. - (6 pts) Brown to black discoloration of flesh.
C. Advanced - (7 pts) Caving in of the flesh and tissues of eyes and throat. - (8 pts) Moist decomposition with bone exposure less than one half that of the area being scored. - (9 pts) Mummification with bone exposure less than one half that of the area being scored.
D. Skeletonisation - (10 pts) Bone exposure of more than half of the area being scored with greasy substances and decomposed tissue. - (11 pts) Bone exposure of more than half the area being scored with desiccated or mummified tissue. - (12 pts) Bones largely dry, but retaining some grease. - (13 pts) Dry bone.
**Categories and Stages of Decomposition for the Torso**
A. Fresh - (1 pt) Fresh, no discolortion
B. Early decomposition - (2 pts) Pink-white appearance with skin slippage and marbling present. - (3 pts) Gray to green discoloration: some flesh relatively fresh. - (4 pts) Bloating with green discoloration and purging of decompositional fluids. - (5 pts) Post-bloating following release of the abdominal gases, with discoloration changing from green to black.
C. Advanced - (2 pts) Pink-white appearance with skin slippage and marbling present. - (3 pts) Gray to green discoloration: some flesh relatively fresh. - (4 pts) Bloating with green discoloration and purging of decompositional fluids. - (5 pts) Post-bloating following release of the abdominal gases, with discoloration changing from green to black.
D. Skeletonisation - (2 pts) Pink-white appearance with skin slippage and marbling present. - (3 pts) Gray to green discoloration: some flesh relatively fresh. - (4 pts) Bloating with green discoloration and purging of decompositional fluids. - (5 pts) Post-bloating following release of the abdominal gases, with discoloration changing from green to black.
**Categories and Stages of Decomposition for the Limbs**
A. Fresh - (1 pt) Fresh, no discolortion
B. Early decomposition - (2 pts) Pink-white appearance with skin slippage of hands and/or feet. - (3 pts) Gray to green discoloration; marbling; some flesh still relatively fresh. - (4 pts) Discoloration and/or brownish shades particularly at edges, drying of fingers, toes, and other projecting extremities. - (5 pts) Brown to black discoloration, skin having a leathery appearance.
C. Advanced - (6 pts) Moist decomposition with bone exposure less than one half that of the area being scored. - (7 pts) Mummification with bone exposure of less than one half that of the area being scored.
D. Skeletonisation - (8 pts) Bone exposure over one half the area being scored, some decomposed tissue and body fluids remaining. - (9 pts) Bones largely dry, but retaining some grease. - (10 pts) Dry bone.

**Table 2:** Results of the linear regression. The correlation, the Residual Sum of Squares, and the slope can be found in the table. The asterisks indicates that the decomposition rate of this weight category was higher compared to the other weight category in the same season.

Weight Category	Season	Correlation	Residual Sum of Squares	Slope
1 (800–2700 g)	Summer	0.656	7745.968	0.225
	Autumn *	0.796	9443.602	0.271
	Winter *	0.877	5750.624	0.115
	Spring *	0.840	6476.971	0.278
2 (4230–10,500 g)	Summer *	0.740	5816.077	0.264
	Autumn	0.513	12,270.024	0.042
	Winter	0.862	4391.745	0.097
	Spring	0.911	2504.902	0.228

**Table 3:** Results of the linear regression on data excluding the plateau phase. The correlation, the Residual Sum of Squares, and the slope can be found in the table, as well as the average maximum TBS reached excluding the plateau phase. The asterisks indicates that the decomposition rate of this weight category was higher compared to the other weight category in the same season.

Weight Category	Season	Average Maximum Reached TBS Excluding Plateau Phase	Correlation	Residual Sum of Squares	Slope
1 (800–2700 g)	Summer	29	0.669	5743.8	0.261
	Autumn *	30	0.816	7088.1	0.332
	Winter *	28	0.877	5750.6	0.115
	Spring *	28	0.858	4956.8	0.294
2 (4230–10,500 g)	Summer *	28	0.747	4068.6	0.316
	Autumn	25	0.516	11,992.7	0.042
	Winter	26	0.862	4391.8	0.097
	Spring	23	0.908	2263.9	0.240

**Table 4:** Overview of the length of time the cadavers were in the bloating stage. The table shows, per weight category, the number of days on average and per season over a period of four years the cadavers were in the bloating stage, including a SEM, and the minimum and maximum days the cadavers were in the bloating stage.

Weight Category	Season	Average (Days)	SEM	Minimum (Days)	Maximum (Days)
1	Summer	1	0.263	1	2
	Autumn	1	0.398	1	3
	Winter	7	0.385	1	25
	Spring	3	1.150	1	10
2	Summer	4	0.780	3	7
	Autumn	21	10.873	2	87
	Winter	11	3.020	2	20
	Spring	8	1.711	4	15

## Data Availability

Data sharing not applicable.

## Supplementary Materials

The following supporting information can be downloaded at: https://www.mdpi.com/article/10.3390/biology12091164/s1, Table S1: Search details finding-based systematic review; Section S2: Review findings; Figure S1: Decomposition rate; Figure S2: Reproducibility; Table S2: Results linear regression including plateau phase; Table S3: Results linear regression excluding plateau phase.
