# Supplementary material for: Subaerial Decomposition of Small-Sized Remains in The Netherlands: Important Findings Regarding the PMI of a Four-Year Taphonomic Study
Source: Biology (Basel). 2023 Aug 24;12(9):1164. doi: 10.3390/biology12091164 (PMC10525113; doi:10.3390/biology12091164)
Supplement: Supplementary file 1 [file biology-12-01164-s001.zip › biology-2544334-supplementary.pdf]

Supplementary Materials

Subaerial Decomposition of Small-Sized Remains in The Netherlands: Important Findings Regarding the PMI of a Four-Year Taphonomic Study

Iris Sluis [1,2,*], Wilma Duijst [1,3] and Tristan Krap [1,2]

[1] Faculty of Law and Criminology, Maastricht University, Minderbroedersberg 4-6, 6211 LK Maastricht, The Netherlands; wilma.duijst@maastrichtuniversity.nl (W.D.); t.krap@maastrichtuniversity.nl (T.K.)
[2] Forensic Laboratory Research, University of Applied Sciences Van Hall Larenstein, Agora 1, 8934 CJ Leeuwarden, The Netherlands
[3] GGD IJsselland, Zeven Alleetjes 1, 8011 CV Zwolle, The Netherlands
* Correspondence: i.sluis@maastrichtuniversity.nl

1. Online Resource: Search terms and references per subchapter

A systematic literature review was performed to link all interesting findings back to the relevant literature. The findings were initially interesting, as they diverged from what is described in the generic literature, which was covered in the introduction of this article. The literature was therefore systematically searched for relevant studies to investigate whether the findings revealed in this study were also reflected in other contexts. In this way, it could be determined whether the findings are specific to small cadavers and/or the Western European mainland. Electronic databases were used for this purpose, namely, PubMed and ScienceDirect. There was no recording year limit (the search included all articles until October 2022), and the language of the articles was limited to English. In the case where insufficient literature on a specific topic was found in the search of the ScienceDirect and PubMed databases, the literature search was extended to Google Scholar. In this study, Google Scholar was used for one finding, namely, the finding about the invertebrate activity. Keywords were specific to each finding and can be found together with the resources in the Table S1, and included combinations of ‘piglet decomposition’, ‘human decomposition’, ‘post-mortem interval’, ‘seasonal decomposition’, ‘freeze-thaw cycles’, ‘decomposition rate’, ‘reproducibility’, ‘body weight’, ‘post-mortem movement’, ‘rainfall’, ‘delayed bloating’, ‘insect activity’, and ‘maggot activity’. Articles were only included if they contained surface or subaerial decomposition; underwater decomposition or indoor decomposition were excluded by title review. The articles were first selected by title, then by abstract, and, after that, the full text was reviewed. After this, the articles were excluded or included in this study. By using the 'snowball method’, the bibliographies of the articles already found were used to obtain additional literature. This was performed by reading an article that was already found with the finding-based systematic search, checking for articles of interest, after which the reference could be obtained via the bibliography. These references were then selected in the same way as described above. In total, as can be seen in Table 1, there were 3170 hits based on the search terms. After the title inclusion, 252 articles remained. After the abstract inclusion, 197 articles were left, which were then extensively reviewed. From the bibliographies of the articles that were already found, to which the ‘snowball method’ was applied, seven articles were found, reviewed, and used. In total, 56 articles were included.

1.1. Search Terms:

Table S1. Search details for the finding-based systematic review

Search term	Hits	Title inclusion	Abstract inclusion
**PubMed**			
Body mass and decomposition	436	8	5
Post-mortem movement and decomposition	20	1	1
Rainfall, insect activity and decomposition	10	2	2
Accumulated degree days and post-mortem interval	81	26	26
Decomposition seasonality post-mortem interval	75	22	20
Piglet and human decomposition	12	0	0
Inter-observer error decomposition	4	1	1
Temperature decomposition post-mortem interval	120	34	31
Human decomposition and post-mortem interval	93	21	17
Post-mortem interval and body weight	204	6	6
Decomposition slugs	31	1	0
Post-mortem interval sus scrofa domesticus	18	4	4
Neonaticide and infanticide Europe	338	24	10
Genomic diversity pig (sus scrofa) and human	99	1	1
**ScienceDirect**			
Decomposition freeze-thaw cycles post-mortem interval	82	3	2
Piglet decomposition body weight post-mortem interval	37	4	4
Rainfall, insect activity and post-mortem interval	119	15	12
Accumulated degree days, decomposition and post-mortem interval	359	21	17
Decomposition seasonality post-mortem interval	409	12	11
Post-mortem interval decomposition effect of snails	35	7	4
Post-mortem interval decomposition snails	48	7	4
Post-mortem interval delayed bloating	44	18	10
Neonaticide and infanticide Europe	41	5	0
Genomic diversity pig (sus scrofa) and human	447	1	1
**Google Scholar**	1	1	1
**'Snowball effect'**	7	7	7
**Total**	**3170**	**252**	**197**

1.2. References per subchapter:

Introduction

1. Alfsdotter, C., & Petaros, A. (2021). Outdoor human decomposition in Sweden: A retrospective quantitative study of forensic-taphonomic changes and postmortem interval in terrestrial and aquatic settings. *Journal of Forensic Sciences*.
2. Almulhim, A. M., & Menezes, R. G. (2020). Evaluation of postmortem changes. In: StatPearls. Treasure Island (FL): StatPearls Publishing; 2022 Jan. 2022 May 8.
3. Baigent, C., Agan, C., Connor, M., & Hansen, E. S. (2020). Autopsy as a form of evisceration: Implications for decomposition rate, pattern, and estimation of postmortem interval. *Forensic science international*, *306*, 110068.
4. Bates, L. N., & Wescott, D. J. (2016). Comparison of decomposition rates between autopsied and non-autopsied human remains. *Forensic science international*, *261*, 93-100.
5. Beauthier, J. P. (2007). Forensic approach of infanticide and neonatal death. *Revue medicale de Bruxelles*, *28*(5), 431-438.
6. Campobasso, C. P., Di Vella, G., & Introna, F. (2001). Factors affecting decomposition and Diptera colonization. *Forensic science international*, *120*(1-2), 18-27.
7. Dabbs, G. R. (2010). Caution! All data are not created equal: The hazards of using National Weather Service data for calculating accumulated degree days. *Forensic science international*, *202*(1-3), e49-e52.
8. Collins, S., Maestrini, L., Ueland, M., & Stuart, B. (2022). A preliminary investigation to determine the suitability of pigs as human analogues for post-mortem lipid analysis. *Talanta Open*, *5*, 100100.
9. Dawson, B. M., Barton, P. S., & Wallman, J. F. (2020). Contrasting insect activity and decomposition of pigs and humans in an Australian environment: A preliminary study. *Forensic science international*, *316*, 110515.
10. Forger, L. V., Woolf, M. S., Simmons, T. L., Swall, J. L., & Singh, B. (2019). A eukaryotic community succession based method for postmortem interval (PMI) estimation of decomposing porcine remains. *Forensic science international*, *302*, 109838.
11. Galloway, A., Birkby, W. H., Jones, A. M., Henry, T. E., & Parks, B. O. (1989). Decay rates of human remains in an arid environment. *Journal of Forensic Science*, *34*(3), 607-616.
12. García-Rojo, A. M., Martínez-Sánchez, A., López, R., de la Vega, J. G., Rica, M., González, M., & Disney, R. H. L. (2013). A mathematical model applied for assisting the estimation of PMI in a case of forensic importance. First record of Conicera similis (Diptera: Phoridae) in a corpse. *Forensic science international*, *231*(1-3), e11-e18.
13. Gelderman, H. T., Boer, L., Naujocks, T., IJzermans, A. C. M., & Duijst, W. L. J. M. (2018). The development of a post-mortem interval estimation for human remains found on land in the Netherlands. *International journal of legal medicine*, *132*(3), 863-873.
14. Gelderman, T., Stigter, E., Krap, T., Amendt, J., & Duijst, W. (2021). The time of death in Dutch court; using the Daubert criteria to evaluate methods to estimate the PMI used in court. *Legal Medicine*, *53*, 101970.
15. Goff, M. L. (2009). Early post-mortem changes and stages of decomposition in exposed cadavers. *Experimental and applied acarology*, *49*(1), 21-36.
16. Giles, S. B., Errickson, D., & Márquez-Grant, N. (2022). A retrospective comparative study to evaluate the reliability of post-mortem interval sources in UK and US medico-legal death investigations. *Science & Justice*, *62*(2), 246-261.

17. Giles, S. B., Errickson, D., & Márquez-Grant, N. (2022). Decomposition variability between the scene and autopsy examination and implications for post-mortem interval estimations. *Journal of Forensic and Legal Medicine*, *85*, 102292.
18. Jeong, S. J., Park, S. H., Park, J. E., Park, S. H., Moon, T. Y., Shin, S. E., & Lee, J. W. (2020). Extended model for estimation of ambient temperature for postmortem interval (PMI) in Korea. *Forensic science international*, *309*, 110196.
19. Kelly, B. D. (2007). Murder, mercury, mental illness: infanticide in nineteenth-century Ireland. *Irish journal of medical science*, *176*(3), 149-152.
20. Kõrgesaar, K., Jordana, X., Gallego, G., Defez, J., & Galtés, I. (2022). Taphonomic model of decomposition. *Legal Medicine*, *56*, 102031.
21. Marhoff, S. J., Fahey, P., Forbes, S. L., & Green, H. (2016). Estimating post-mortem interval using accumulated degree-days and a degree of decomposition index in Australia: a validation study. *Australian Journal of Forensic Sciences*, *48*(1), 24-36.
22. Marcikić, M., Dumenčić, B., Matuzalem, E., Marjanović, K., Požgain, I., & Ugljarević, M. (2006). Infanticide in eastern Croatia. *Collegium antropologicum*, *30*(2), 437-442.
23. Marks, M. N., & Kumar, R. (1993). Infanticide in England and Wales. *Medicine, Science and the Law*, *33*(4), 329-339.
24. Marks, M. N., & Kumar, R. (1996). Infanticide in Scotland. *Medicine, Science and the Law*, *36*(4), 299-305.
25. Matuszewski, S., Hall, M. J., Moreau, G., Schoenly, K. G., Tarone, A. M., & Villet, M. H. (2020). Pigs vs people: the use of pigs as analogues for humans in forensic entomology and taphonomy research. *International journal of legal medicine*, *134*(2), 793-810.
26. Megyesi, M. S., Nawrocki, S. P., & Haskell, N. H. (2005). Using accumulated degree-days to estimate the postmortem interval from decomposed human remains. *Journal of Forensic Science*, *50*(3), 1-9.
27. Michaud, J. P., & Moreau, G. (2011). A statistical approach based on accumulated degree-days to predict decomposition-related processes in forensic studies. *Journal of forensic sciences*, *56*(1), 229-232.
28. Moffatt, C., Simmons, T., & Lynch-Aird, J. (2016). An improved equation for TBS and ADD: establishing a reliable postmortem interval framework for casework and experimental studies. *Journal of Forensic Sciences*, *61*, S201-S207.
29. Myburgh, J., L'Abbé, E. N., Steyn, M., & Becker, P. J. (2013). Estimating the postmortem interval (PMI) using accumulated degree-days (ADD) in a temperate region of South Africa. *Forensic science international*, *229*(1-3), 165-e1.
30. Onyejike, D. N., Esomonu, U. G., Fischer, V. A., Onyejike, I. M., Akukwu, D. C., Obiesie, I. J., ... & Obun, C. (2022). Factors that influence the estimation of post mortem interval in a Guinea forest-savannah vegetation of Nigeria. *Forensic Science International: Reports*, 100278.
31. Putkonen, H., Collander, J., Weizmann-Henelius, G., & Eronen, M. (2007). Legal outcomes of all suspected neonaticides in Finland 1980–2000. *International journal of law and psychiatry*, *30*(3), 248-254.
32. Putkonen, H., Weizmann-Henelius, G., Collander, J., Santtila, P., & Eronen, M. (2007). Neonaticides may be more preventable and heterogeneous than previously thought–neonaticides in Finland 1980–2000. *Archives of Women's Mental Health*, *10*(1), 15-23.
33. Ribéreau-Gayon, A., Rando, C., Morgan, R. M., & Carter, D. O. (2018). The suitability of visual taphonomic methods for digital photographs: An experimental approach with pig carcasses in a tropical climate. *Science & Justice*, *58*(3), 167-176.

34. Röglin, A., Szentiks, C. A., Dreßler, J., Ondruschka, B., & Schwarz, M. (2022). Entomological identification of the post-mortem colonization of wolf cadavers in different decomposition stages. *Science & Justice*, *62*(5), 520-529.
35. Scott, R. (2020). Infanticide and Infanticide Statutes in Australia and New Zealand. *Journal of law and medicine*, *27*(4), 1014-1046.
36. Simmons, T., Adlam, R. E., & Moffatt, C. (2010). Debugging decomposition data—comparative taphonomic studies and the influence of insects and carcass size on decomposition rate. *Journal of forensic sciences*, *55*(1), 8-13.
37. Smith, D. H., Ehrett, C., Weisensee, K., & Tica, C. (2022). Commentary on: Megyesi MS, Nawrocki SP, Haskell NH. Using accumulated degree-days to estimate the postmortem interval from decomposed human remains. J Forensic Sci. 2005; 50 (3): 618–26. doi: 10.1520/JFS2004017; and Moffatt C,
38. Simmons T, Lynch-Aird J. An improved equation for TBS and ADD: Establishing a reliable postmortem interval framework for casework and experimental studies. J Forensic Sci. 2016; 61 (Suppl 1): S201–S207. doi: 10.1111/1556-4029.12931. *Journal of Forensic Sciences*.
39. Suckling, J. K., Spradley, M. K., & Godde, K. (2016). A longitudinal study on human outdoor decomposition in Central Texas. *Journal of forensic sciences*, *61*(1), 19-25.
40. Tanaka, C. T., Berger, W., Valença, A. M., Coutinho, E. S., Jean-Louis, G., Fontenelle, L. F., & Mendlowicz, M. V. (2017). The worldwide incidence of neonaticide: a systematic review. *Archives of women's mental health*, *20*(2), 249-256.
41. Tursz, A., & Cook, J. M. (2011). A population-based survey of neonaticides using judicial data. *Archives of Disease in Childhood-Fetal and Neonatal Edition*, *96*(4), F259-F263.
42. Zhu, W. H., Yang, M. Z., Zheng, Z., Sun, K., & Mo, Y. N. (2021). Research Progress on Accumulated Degree Days for PMI Estimation. *Fa yi xue za zhi*, *37*(3), 396-401.

Decomposition rate

1. Adlam, R. E., & Simmons, T. (2007). The effect of repeated physical disturbance on soft tissue decomposition—are taphonomic studies an accurate reflection of decomposition?. *Journal of Forensic Sciences*, *52*(5), 1007-1014.
2. Alfsdotter, C., & Petaros, A. (2021). Outdoor human decomposition in Sweden: A retrospective quantitative study of forensic-taphonomic changes and postmortem interval in terrestrial and aquatic settings. *Journal of Forensic Sciences*.
3. Almulhim, A. M., & Menezes, R. G. (2020). Evaluation of postmortem changes.
4. Battán Horenstein, M., Xavier Linhares, A., Rosso de Ferradas, B., & García, D. D. (2010). Decomposition and dipteran succession in pig carrion in central Argentina: ecological aspects and their importance in forensic science. *Medical and Veterinary Entomology*, *24*(1), 16-25.
5. Belk, A. D., Deel, H. L., Burcham, Z. M., Knight, R., Carter, D. O., & Metcalf, J. L. (2018). Animal models for understanding microbial decomposition of human remains. *Drug Discovery Today: Disease Models*, *28*, 117-125.
6. Brooks, J. W. (2016). Postmortem changes in animal carcasses and estimation of the postmortem interval. *Veterinary Pathology*, *53*(5), 929-940.
7. e Castro, C. P., García, M. D., da Silva, P. M., e Silva, I. F., & Serrano, A. (2013). Coleoptera of forensic interest: a study of seasonal community composition and succession in Lisbon, Portugal. *Forensic science international*, *232*(1-3), 73-83.
8. Campobasso, C. P., Di Vella, G., & Introna, F. (2001). Factors affecting decomposition and Diptera colonization. *Forensic science international*, *120*(1-2), 18-27.
9. Cockle, D. L., & Bell, L. S. (2015). Human decomposition and the reliability of a 'Universal'model for post mortem interval estimations. *Forensic science international*, *253*, 136-e1.

10. Dabbs, G. R. (2015). How should forensic anthropologists correct national weather service temperature data for use in estimating the postmortem interval?. *Journal of forensic sciences*, *60*(3), 581-587.
11. Finaughty, D. A., & Morris, A. G. (2019). Precocious natural mummification in a temperate climate (Western Cape, South Africa). *Forensic science international*, *303*, 109948.
12. Ferreira, M. T., & Cunha, E. (2013). Can we infer post mortem interval on the basis of decomposition rate? A case from a Portuguese cemetery. *Forensic science international*, *226*(1-3), 298-e1.
13. Gelderman, H. T., Kruiver, C. A., Oostra, R. J., Zeegers, M. P., & Duijst, W. L. J. M. (2019). Estimation of the postmortem interval based on the human decomposition process. *Journal of forensic and legal medicine*, *61*, 122-127.
14. Giles, S. B., Harrison, K., Errickson, D., & Márquez-Grant, N. (2020). The effect of seasonality on the application of accumulated degree-days to estimate the early post-mortem interval. *Forensic Science International*, *315*, 110419.
15. Guebelin, D. L., Dobay, A., Ebert, L., Betschart, E., Thali, M. J., & Franckenberg, S. (2021). Correlation of age, sex and season with the state of human decomposition as quantified by postmortem computed tomography. *Forensic Science, Medicine and Pathology*, *17*(2), 185-191.
16. Iancu, L., Carter, D. O., Junkins, E. N., & Purcarea, C. (2015). Using bacterial and necrophagous insect dynamics for post-mortem interval estimation during cold season: Novel case study in Romania. *Forensic science international*, *254*, 106-117.
17. Iancu, L., Dean, D. E., & Purcarea, C. (2018). Temperature influence on prevailing necrophagous diptera and bacterial taxa with forensic implications for postmortem interval estimation: A review. *Journal of medical entomology*, *55*(6), 1369-1379.
18. Komar, D. A. (1998). Decay rates in a cold climate region: a review of cases involving advanced decomposition from the Medical Examiner's Office in Edmonton, Alberta. *Journal of Forensic Science*, *43*(1), 57-61.
19. Krajčovič, J., Janík, M., Novomeský, F., Straka, Ľ., & Hejna, P. (2014). Feasibility, diagnostic validity and limits of postmortem evaluation of a newborn infant following an extremely prolonged freezing interval: A thanatological case study. *Legal Medicine*, *16*(6), 376-380.
20. Maile, A. E., Inoue, C. G., Barksdale, L. E., & Carter, D. O. (2017). Toward a universal equation to estimate postmortem interval. *Forensic science international*, *272*, 150-153.
21. Marais-Werner, A., Myburgh, J., Becker, P. J., & Steyn, M. (2018). A comparison between decomposition rates of buried and surface remains in a temperate region of South Africa. *International journal of legal medicine*, *132*(1), 301-309.
22. Marhoff-Beard, S. J., Forbes, S. L., & Green, H. (2018). The validation of 'universal'PMI methods for the estimation of time since death in temperate Australian climates. *Forensic science international*, *291*, 158-166.
23. Meyer, J., Anderson, B., & Carter, D. O. (2013). Seasonal variation of carcass decomposition and gravesoil chemistry in a cold (Dfa) climate. *Journal of forensic sciences*, *58*(5), 1175-1182.
24. Micozzi, M. S. (1997). Frozen environments and soft tissue preservation. *Forensic taphonomy: the postmortem fate of human remains*, 171-180.
25. Nguyễn, N. H., Dương, M. T., Trần, T. N., Phạm, P. T., Grottke, O., Tolba, R., & Staat, M. (2012). Influence of a freeze–thaw cycle on the stress–stretch curves of tissues of porcine abdominal organs. *Journal of biomechanics*, *45*(14), 2382-2386.
26. Ries, A. C. R., Costa-Silva, V., Dos Santos, C. F., Blochtein, B., & Thyssen, P. J. (2021). Factors Affecting the Composition and Succession of Beetles in Exposed Pig Carcasses in Southern Brazil. *Journal of Medical Entomology*, *58*(1), 104-113.

27. Roberts, L. G., & Dabbs, G. R. (2015). A Taphonomic Study Exploring the Differences in Decomposition Rate and Manner between Frozen and Never Frozen Domestic Pigs (S us scrofa). *Journal of forensic sciences*, *60*(3), 588-594.
28. Schoenly, K., Griest, K., & Rhine, S. (1991). An experimental field protocol for investigating the postmortem interval using multidisciplinary indicators. *Journal of Forensic Science*, *36*(5), 1395-1415.
29. Varlet, V., Joye, C., Forbes, S. L., & Grabherr, S. (2020). Revolution in death sciences: body farms and taphonomics blooming. A review investigating the advantages, ethical and legal aspects in a Swiss context. *International journal of legal medicine*, *134*, 1875-1895.
30. Vass, A. A. (2011). The elusive universal post-mortem interval formula. *Forensic science international*, *204*(1-3), 34-40.

<u>Reproducibility</u>

1. Dabbs, G. R., Connor, M., & Bytheway, J. A. (2016). Interobserver reliability of the total body score system for quantifying human decomposition. *Journal of forensic sciences*, *61*(2), 445-451.
2. Dautartas, A., Kenyhercz, M. W., Vidoli, G. M., Meadows Jantz, L., Mundorff, A., & Steadman, D. W. (2018). Differential decomposition among pig, rabbit, and human remains. *Journal of forensic sciences*, *63*(6), 1673-1683.
3. Forbes, M. N., Finaughty, D. A., Miles, K. L., & Gibbon, V. E. (2019). Inaccuracy of accumulated degree day models for estimating terrestrial post-mortem intervals in Cape Town, South Africa. *Forensic science international*, *296*, 67-73.
4. Keough, N., Myburgh, J., & Steyn, M. (2017). Scoring of decomposition: a proposed amendment to the method when using a pig model for human studies. *Journal of forensic sciences*, *62*(4), 986-993.
5. Zhang, C., & Plastow, G. (2011). Genomic Diversity in Pig (Sus scrofa) and its Comparison with Human and other Livestock. *Current Genomics*, *12*(2), 138-146.

<u>Body weight</u>

1. Barton, P. S., Dawson, B. M., Barton, A. F., Joshua, S., & Wallman, J. F. (2021). Temperature dynamics in different body regions of decomposing vertebrate remains. *Forensic Science International*, *325*, 110900.
2. Campobasso, C. P., Di Vella, G., & Introna, F. (2001). Factors affecting decomposition and Diptera colonization. *Forensic science international*, *120*(1-2), 18-27.
3. Cordeiro, C., Ordóñez-Mayán, L., Lendoiro, E., Febrero-Bande, M., Vieira, D. N., & Muñoz-Barús, J. I. (2019). A reliable method for estimating the postmortem interval from the biochemistry of the vitreous humor, temperature and body weight. *Forensic science international*, *295*, 157-168.
4. Dawson, B. M., Wallman, J. F., & Barton, P. S. (2022). How does mass loss compare with total body score when assessing decomposition of human and pig cadavers?. *Forensic Science, Medicine and Pathology*, 1-9.
5. Hewadikaram, K. A., & Goff, M. L. (1991). Effect of carcass size on rate of decomposition and arthropod succession patterns. *The American Journal of Forensic Medicine and Pathology*, *12*(3), 235-240.
6. Mann, R. W., Bass, W. M., & Meadows, L. (1990). Time since death and decomposition of the human body: variables and observations in case and experimental field studies. *Journal of Forensic Science*, *35*(1), 103-111.

7. Matuszewski, S., Bajerlein, D., Konwerski, S., & Szpila, K. (2010). Insect succession and carrion decomposition in selected forests of Central Europe. Part 1: Pattern and rate of decomposition. *Forensic Science International*, *194*(1-3), 85-93.
8. Matuszewski, S., Konwerski, S., Frątczak, K., & Szafałowicz, M. (2014). Effect of body mass and clothing on decomposition of pig carcasses. *International journal of legal medicine*, *128*(6), 1039-1048.
9. Matuszewski, S., Frątczak, K., Konwerski, S., Bajerlein, D., Szpila, K., Jarmusz, M., ... & Mądra, A. (2016). Effect of body mass and clothing on carrion entomofauna. *International journal of legal medicine*, *130*(1), 221-232.
10. Miles, K. L., Finaughty, D. A., & Gibbon, V. E. (2020). A review of experimental design in forensic taphonomy: moving towards forensic realism. *Forensic Sciences Research*, *5*(4), 249-259.
11. Notter, S. J., Stuart, B. H., Rowe, R., & Langlois, N. (2009). The initial changes of fat deposits during the decomposition of human and pig remains. *Journal of Forensic Sciences*, *54*(1), 195-201.
12. Olakanye, A. O., Nelson, A., & Ralebitso-Senior, T. K. (2017). A comparative in situ decomposition study using still born piglets and leaf litter from a deciduous forest. *Forensic science international*, *276*, 85-92.
13. Roberts, L. G., Spencer, J. R., & Dabbs, G. R. (2017). The effect of body mass on outdoor adult human decomposition. *Journal of forensic sciences*, *62*(5), 1145-1150.
14. Rosier, E., Loix, S., Develter, W., Van de Voorde, W., Cuypers, E., & Tytgat, J. (2017). Differentiation between decomposed remains of human origin and bigger mammals. *Journal of forensic and legal medicine*, *50*, 28-35.
15. Ross, A. H., & Hale, A. R. (2018). Decomposition of juvenile-sized remains: a macro-and microscopic perspective. *Forensic sciences research*, *3*(4), 310-319.
16. Salimi, M., Chatrabgoun, O., Akbarzadeh, K., Oshaghi, M., Falahati, M. H., Rafizadeh, S., ... & Rassi, Y. (2018). Evaluation of insect succession patterns and carcass weight loss for the estimation of postmortem interval. *Journal of medical entomology*, *55*(6), 1410-1422.
17. Sharma, R., Garg, R. K., & Gaur, J. R. (2015). Various methods for the estimation of the post mortem interval from Calliphoridae: A review. *Egyptian Journal of Forensic Sciences*, *5*(1), 1-12.
18. Sutherland, A., Myburgh, J., Steyn, M., & Becker, P. J. (2013). The effect of body size on the rate of decomposition in a temperate region of South Africa. *Forensic science international*, *231*(1-3), 257-262.
19. Zhou, C., & Byard, R. W. (2011). Factors and processes causing accelerated decomposition in human cadavers–an overview. *Journal of forensic and legal medicine*, *18*(1), 6-9.

<u>Post-mortem movement</u>

1. Sharma, R., Garg, R. K., & Gaur, J. R. (2015). Various methods for the estimation of the post mortem interval from Calliphoridae: A review. *Egyptian Journal of Forensic Sciences*, *5*(1), 1-12.
2. Wilson, A., Serafin, S., Seckiner, D., Berry, R., & Mallett, X. (2019). Evaluating the utility of time-lapse imaging in the estimation of post-mortem interval: an Australian case study. *Forensic Science International: Synergy*, *1*, 204-210.
3. Wilson, A., Neilsen, P., Berry, R., Seckiner, D., & Mallett, X. (2020). Quantifying human post-mortem movement resultant from decomposition processes. *Forensic Science International: Synergy*, *2*, 248-261.

<u>Rainfall</u>

1. Archer, M. S. (2004). Rainfall and temperature effects on the decomposition rate of exposed neonatal remains. *Science & justice: journal of the Forensic Science Society*, *44*(1), 35-41.

2. Campobasso, C. P., Di Vella, G., & Introna, F. (2001). Factors affecting decomposition and Diptera colonization. *Forensic science international*, *120*(1-2), 18-27.
3. de Carvalho, L. M. L., & Linhares, A. X. (2001). Seasonality of insect succession and pig carcass decomposition in a natural forest area in southeastern Brazil. *Journal of forensic science*, *46*(3), 604-608.
4. Cockle, D. L., & Bell, L. S. (2017). The environmental variables that impact human decomposition in terrestrially exposed contexts within Canada. *Science & Justice*, *57*(2), 107-117.
5. Cogswell, G. C., & Cross, P. A. (2021). The effects of surface variation on the decomposition of pig carcasses. *Journal of Forensic and Legal Medicine*, *79*, 102108.
6. Mahat, N. A., Zafarina, Z., & Jayaprakash, P. T. (2009). Influence of rain and malathion on the oviposition and development of blowflies (Diptera: Calliphoridae) infesting rabbit carcasses in Kelantan, Malaysia. *Forensic science international*, *192*(1-3), 19-28.
7. Lyu, Z., Wan, L. H., Yang, Y. Q., Tang, R., & Xu, L. Z. (2016). A checklist of beetles (Insecta, Coleoptera) on pig carcasses in the suburban area of southwestern China: A preliminary study and its forensic relevance. *Journal of forensic and legal medicine*, *41*, 42-48.
8. Sharanowski, B. J., Walker, E. G., & Anderson, G. S. (2008). Insect succession and decomposition patterns on shaded and sunlit carrion in Saskatchewan in three different seasons. *Forensic science international*, *179*(2-3), 219-240.
9. Tembe, D., & Mukaratirwa, S. (2021). Insect Succession and Decomposition Pattern on Pig Carrion During Warm and Cold Seasons in Kwazulu-Natal Province of South Africa. *Journal of Medical Entomology*.
10. Voss, S. C., Cook, D. F., & Dadour, I. R. (2011). Decomposition and insect succession of clothed and unclothed carcasses in Western Australia. *Forensic Science International*, *211*(1-3), 67-75.

<u>Delayed bloating</u>

1. Campobasso, C. P., Di Vella, G., & Introna, F. (2001). Factors affecting decomposition and Diptera colonization. *Forensic science international*, *120*(1-2), 18-27.
2. Collis, S., & Johnson, C. P. (2019). The decomposed cadaver. *Diagnostic Histopathology*, *25*(11), 431-435.
3. Díaz-Aranda, L. M., Martín-Vega, D., Gómez-Gómez, A., Cifrián, B., & Baz, A. (2018). Annual variation in decomposition and insect succession at a periurban area of central Iberian Peninsula. *Journal of Forensic and Legal Medicine*, *56*, 21-31.
4. Eberhardt, T. L., & Elliot, D. A. (2008). A preliminary investigation of insect colonisation and succession on remains in New Zealand. *Forensic Science International*, *176*(2-3), 217-223.
5. Finaughty, D. A., & Morris, A. G. (2019). Precocious natural mummification in a temperate climate (Western Cape, South Africa). *Forensic science international*, *303*, 109948.
6. Galloway, A., Birkby, W., Jones, A., Henry, T., and Parks, B., 1989. Decay Rates of Human Remains in an Arid Environment. *Journal of Forensic Sciences*, 34(3), 607-16.
7. Giles, S. B., Harrison, K., Errickson, D., & Márquez-Grant, N. (2020). The effect of seasonality on the application of accumulated degree-days to estimate the early post-mortem interval. *Forensic Science International*, *315*, 110419.
8. Giles, S. B., Errickson, D., & Márquez-Grant, N. (2022). Decomposition variability between the scene and autopsy examination and implications for post-mortem interval estimations. *Journal of Forensic and Legal Medicine*, *85*, 102292.
9. Matuszewski, S., Bajerlein, D., Konwerski, S., & Szpila, K. (2010). Insect succession and carrion decomposition in selected forests of Central Europe. Part 1: Pattern and rate of decomposition. *Forensic science international*, *194*(1-3), 85-93.

10. Matuszewski, S., Konwerski, S., Frątczak, K., & Szafałowicz, M., 2014. Effect of body mass and clothing on decomposition of pig carcasses. *International journal of legal medicine*, *128*(6), 1039-1048.
11. Röglin, A., Szentiks, C. A., Dreßler, J., Ondruschka, B., & Schwarz, M. (2022). Entomological identification of the post-mortem colonization of wolf cadavers in different decomposition stages. *Science & Justice*, *62*(5), 520-529.
12. Sharanowski, B. J., Walker, E. G., & Anderson, G. S. (2008). Insect succession and decomposition patterns on shaded and sunlit carrion in Saskatchewan in three different seasons. *Forensic science international*, *179*(2-3), 219-240.

Invertebrate activity

1. Centeno, N., Maldonado, M., & Oliva, A. (2002). Seasonal patterns of arthropods occurring on sheltered and unsheltered pig carcasses in Buenos Aires Province (Argentina). *Forensic Science International*, *126*(1), 63-70.
2. Cogswell, G. C., & Cross, P. A. (2021). The effects of surface variation on the decomposition of pig carcasses. *Journal of Forensic and Legal Medicine*, *79*, 102108.
3. Goff, M. L., 2009. Early post-mortem changes and stages of decomposition in exposed cadavers. *Experimental and applied acarology*, *49*(1), 21-36.
4. Gunn, A. (2016). The colonisation of remains by the muscid flies Muscina stabulans (Fallén) and Muscina prolapsa (Harris)(Diptera: Muscidae). *Forensic science international*, *266*, 349-356.
5. Meyer, J., Anderson, B., & Carter, D. O. (2013). Seasonal variation of carcass decomposition and gravesoil chemistry in a cold (Dfa) climate. *Journal of forensic sciences*, *58*(5), 1175-1182.
6. Moreau, G., Ramal, A. F., Letana, S. D., & Horgan, F. G. (2022). Death in the paddy field: Carcass decomposition and associated arthropods in subunits of a rice field landscape. *Forensic Science International*, *335*, 111288.
7. O'Brien, R. C., Forbes, S. L., Meyer, J., & Dadour, I. (2010). Forensically significant scavenging guilds in the southwest of Western Australia. *Forensic Science International*, *198*(1-3), 85-91.
8. Paczkowski, S., Nicke, S., Ziegenhagen, H., & Schütz, S. (2015). Volatile Emission of Decomposing Pig Carcasses (Sus scrofa domesticus L.) as an Indicator for the P ostmortem Interval. *Journal of Forensic Sciences*, *60*, S130-S137.
9. Probst, C., Gethmann, J., Amendt, J., Lutz, L., Teifke, J. P., & Conraths, F. J. (2020). Estimating the postmortem interval of wild boar carcasses. *Veterinary Sciences*, *7*(1), 6.
10. Šuláková, H., & Barták, M., 2013. Forensically important Calliphoridae (Diptera) associated with animal and human decomposition in the Czech Republic: preliminary results. *Acta Musei Silesiae, Scientiae Naturales*, *62*(3), 255-266.

2. Review findings

2.1. Finding 1: Decomposition rate

2.1.2. Review finding

After the title and abstract selection, 30 articles were of interest for further consideration. These articles were reviewed, of which 6 were eventually used in this subchapter.

Decomposition is a progressive and irreversible process, influenced by many factors. The most important factor is temperature, but when looking at seasonal decomposition, factors such as rainfall and humidity also play important roles [1-2]. An explanation for a relatively lower decomposition rate at colder temperatures can be found in external variables, such as bacterial growth and insect activity, which normally stimulate decomposition but are inhibited by the colder temperatures [1, 3-4]. Cadavers exposed to freeze-thaw cycles appear to be more prone to decompose from the outside to the inside, while cadavers that have not undergone a freeze-thaw cycle are more likely to exhibit the internal ('inside-out') presence of insects and microorganisms [5]. The effects of freezing or cold temperatures should therefore be taken into account as these influence putrefaction and later stages of decomposition. The study by Giles et al. (2020) found that a similar decomposition rate was observed between the summer and autumn cadavers. In addition, the summer and autumn cadavers had highest decay rates in the early post-mortem period. This similarity was explained by the relatively high humidity in these periods compared to the winter and spring periods. This higher humidity increased the decomposition rate by increasing the influence of insect larvae and reducing the chance of mummification [6]. The findings by Giles et al. (2020) are in line with our study, as we also observed higher decomposition rates of summer and autumn cadavers relative to the winter and spring cadavers.

2.2. Finding 2: Reproducibility

After the title and abstract selection, 5 articles were of interest for further consideration. These articles were reviewed, and all 5 articles were eventually used in this subchapter.

The finding that the results are reproducible is important as it increases the reliability of the results. The within-season variation, shown in Figure 4 of the article and Figure 2 of the Supplementary Material, can be explained by intrinsic (weight of the cadavers varied) and extrinsic variables and by an observer variation, as all years were observed by other researchers [7-8]. Research by Dautartas et al. (2018) shows that in decomposition studies with pig cadavers little decomposition variability is observed and that therefore the reproducibility of decomposition studies with pig cadavers is good. The reasons for this are that pigs from a farm are often fed homogeneous diets, have a similar pattern of exposure to certain pathogens, and are equal in body weight [9-10]. Besides, the genetic diversity is smaller and the heterozygosity is relatively lower in pigs than in humans [11]. The intrinsic variables that can influence the decomposition study with pig cadavers are therefore limited.

2.3. Finding 3: Body weight

After the title and abstract selection, 19 articles were of interest for further consideration. These articles were reviewed, of which 7 were eventually used in this sub-chapter.

From the literature, there is no uniformity regarding the effect of weight on decomposition, while the weight for example is an important factor concerning the rate of cooling of the body [12-16]. In this case, a lower body weight reaches the ambient temperature more quickly as opposed to higher body weights [17]. In addition, the biomass and surface-area-to-volume ratio play a role. For larger cadavers, this would be higher, and thus the surface area available for decomposition changes with increased body

size. As a result, a ~20 kg body will not decompose at the same rate or with the same pattern as a ~60 kg body [18]. Sutherland (2013) stated that smaller cadavers (3-35 kg) would decompose 2.82 times faster than larger cadavers (60-90 kg) [19]. In addition, it could be established that more weight loss was noted in the smaller cadavers, especially during advanced decomposition (>7 days) and that the bloating stage in the larger cadavers started earlier and lasted longer, probably due to larger and more complex microbial substances and a slower heat loss. The active decay started in the smaller and larger cadavers at the same time but lasted longer with the larger cadavers [20-21].

2.4. Finding 4: Post-mortem movement

After the title and abstract selection, 3 articles were of interest for further consideration. These articles were reviewed, of which 2 were eventually used in this subchapter.

Post-mortem movement was extensively studied by Wilson et al. (2020). In the study conducted by Wilson and her colleagues, a decomposing human donor was observed for 16 months for post-mortem movement using time-lapse images. This study showed that post-mortem movement occurs in all limbs, taking place the most at the advanced decomposition phase, with the lower extremities moving the most. An explanation for this post-mortem movement was found in the mummification of tissues [22].

2.5. Finding 5: Rainfall

After the title and abstract selection, 10 articles were of interest for further consideration. These articles were reviewed, of which 7 were eventually used in this subchapter.

Since 1980, several studies have been conducted into the decomposition process of corpses (human and piglets). Here the influence of the extrinsic factors, including precipitation, was investigated on the decomposition process and the rate. There is no uniform outcome from the literature regarding the effect of rainfall on the decomposition rate. There are studies stating that rainfall expels insect activity and thereby affecting and delaying the decomposition rate [23-25]. Rain would be a clear deterrent to laying eggs on a body [15, 26]. There are also studies arguing that rainfall does not affect the decomposition rate because insects retreat into body cavities and continue feeding while remaining protected from temperature changes, wind, rain, snow, and solar radiation [1-2, 27] [12, 16, 28]. A study by Archer (2004) found that neonates could even decompose faster when exposed to rain. An explanation for this was that precipitation and soil moisture keep the body wet and this would reduce dehydration. This facilitates maggot activity and bacterial activity, which would accelerate decomposition [28]. However, these studies were all conducted with large cadavers, except for the study by Archer 2004 (2004) and Lyu et al. (2016) who used respectively pig cadavers of ~2 kg and ~3-10 kg). Our study obtained a similar result to the study conducted by Lyu et al. (2016) in China with *Sus scrofa L.*, which also showed a delayed decomposition rate in smaller cadavers, but used a different pig species than we used [24].

2.6. Finding 6: Delayed bloating

After the title and abstract selection, 12 articles were of interest for further consideration. These articles were reviewed, of which 5 were eventually used in this subchapter.

Multiple decomposition studies have been performed where it was observed that the bloated stage was delayed and persisted for an extended period of time. It is known from the generic literature that the bloating stage usually begins 48 hours after death and lasts on average around seven days, with an extreme of 13 days, partly depending on the ambient temperature [1, 23, 29]. In addition to ambient temperature, the size of the cadaver plays a role in its inflation [20]. The study by Matuszewski et al. 2010 found that active decay occurred before the bloating stage had begun. In addition, the season had

a significant effect on the duration of the bloating stage, with a longer duration in spring than in summer and autumn. The study used domestic pig (*Sus scrofa domestica*) cadavers (weight range= 14–39 kg), and decomposition was observed during summer, autumn and spring in Western Poland [30]. Díaz-Aranda et al. 2018 also noted in their seasonal decomposition study in central Spain, using domestic pig (*Sus domesticus Erxleben*) cadavers (weight range=24–32kg), that active decay during the winter and spring months occurred earlier than the bloating stage, and the processes then began to overlap. Also, the bloated stage and active decay in the winter period started later and lasted longer compared to the other seasons. As a result, the advanced decay did not start until day 100-109, and continued through the following season. In the winter period, the bloating stage lasted an average of 20 days, in the spring 17 days, and in the summer and autumn months on average of 2 days [31]. An explanation for delayed bloating in general can be found in a lower ambient temperature that inhibits bacterial proliferation and insect activity. After a period of higher temperatures, they will become active again. However, the build-up of bacteria that then takes place on the inside of the body starts slowly. These bacteria eventually leads to the bloating of the body, but due to this slow build-up, the bloating of the body will occur later than seen in higher ambient temperatures (for example in the summer season) [1]. The delayed bloating of cadavers in the colder seasons in, for example, the winter season, means that cadavers from the spring season in the chronology would already have passed this stage. This results in an incorrect decomposition score and causes an inaccurate PMI estimate, thus a large PMI range.

2.7. Finding 7: Invertebrate activity

After the title and abstract selection, 10 articles were of interest for further consideration. These articles were reviewed, of which 3 were eventually used in this subchapter.

Goff 2009 argues that there are several relationships between arthropods and decomposing bodies. However, this relationship differs between taxa, with not every relationship being of equal value to the study. Smith 1986 described four ecological categories of invertebrates, where a distinction could be made between the necrophages, parasites and predators on the necrophagous, omnivorous and adventive species. Necrophagous species such as the flies (Diptera) and beetles (Coleptera) mainly feed on the cadaver. The parasites and predators on the necrophagous species, such as the wasps (Hymenoptera), feed on the present arthropods on the cadaver and the cadaver itself. In the Diptera, Coleptera and Hymenoptera it also occurs that the larvae are necrophages during the early development of their lives, but become predators at other stages of their development, eating other larvae, making them omnivores. Adventive species include those that use the body as an extension of their normal habitat, such as spiders and centipedes. Slugs are omnivores according to the study by Smith 1986 [32-33]. In the study of Šuláková & Barták 2013, the presence of slugs was found in the decomposition process of both experiments with pig cadavers and the real cases. This mainly occurred between days 2 and 3 post-mortem, with the slugs being mainly present on the ears and lower parts of the abdomen and back. The slug activity was recorded sporadically until the end of the experiment. It was concluded that slugs often occur and participate in the decomposition process, but that the forensic importance here is still unclear, as the connection between the presence of the slugs and the specific stages of decomposition is still missing [33]. The study by Paczkowski et al. 2015 also noted high activity of slugs. These slugs were especially present after regular rainfall, which left the cadaver body moist. The slugs fed on the cadaver as well as the eggs laid on the cadaver by other insects [33-34]. The fact that slugs participate in the decomposition process appears to be very common according to the research of Šuláková & Barták 2013, even though the forensic significance of this is still unclear due to the lack of connection with the specific phase of decomposition [33].

3. Figures and Tables

3.1. Decomposition rate

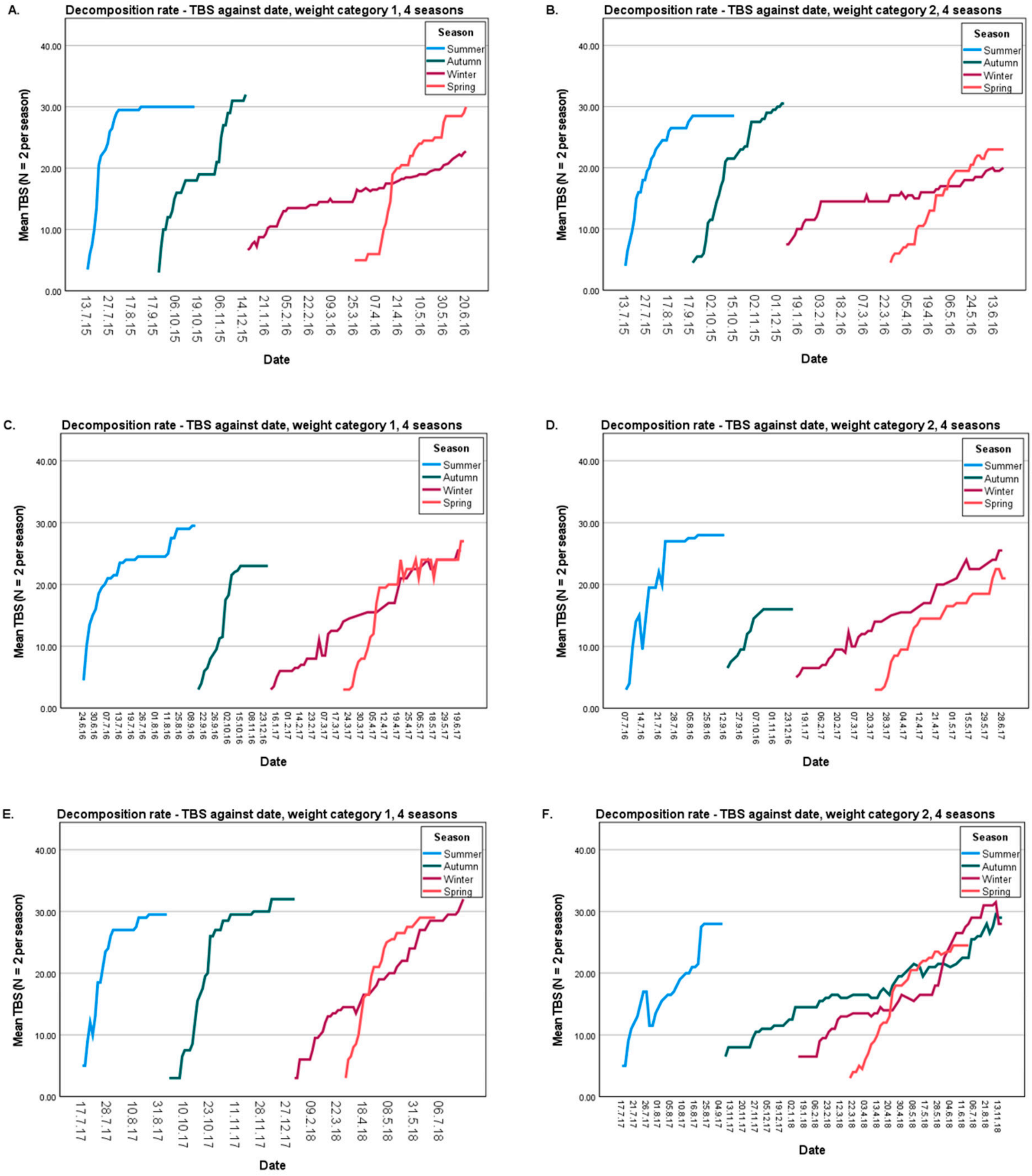

* The decomposition rate of the weight category 2 was slower during autumn of 2017 than during the other years, so it was decided to observe this period longer.

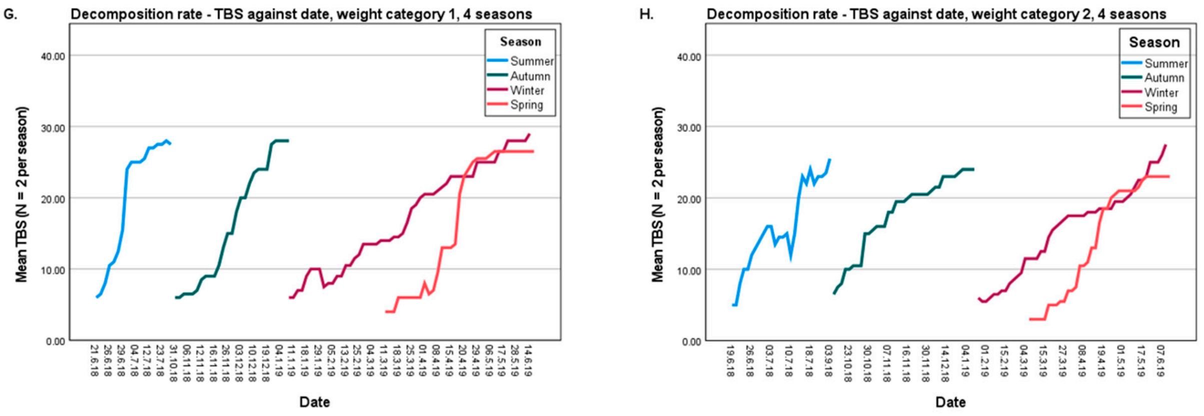

Figure S1. Decomposition rate. Mean Total Body Score (TBS) per season versus time (n = 2 per season). A: research years 2015-2016 weight cat. 1. B: research years 2015-2016 weight cat. 2. C: research years 2016-2017 weight cat. 1. D: research years 2016-2017 weight cat. 2. E: research years 2017-2018 weight cat. 1. F: Research years 2017-2018 weight cat. 2. G: Research years 2018-2019 weight cat. 1. H: Research years 2018-2019 weight cat. 2

3.2. Reproducibility

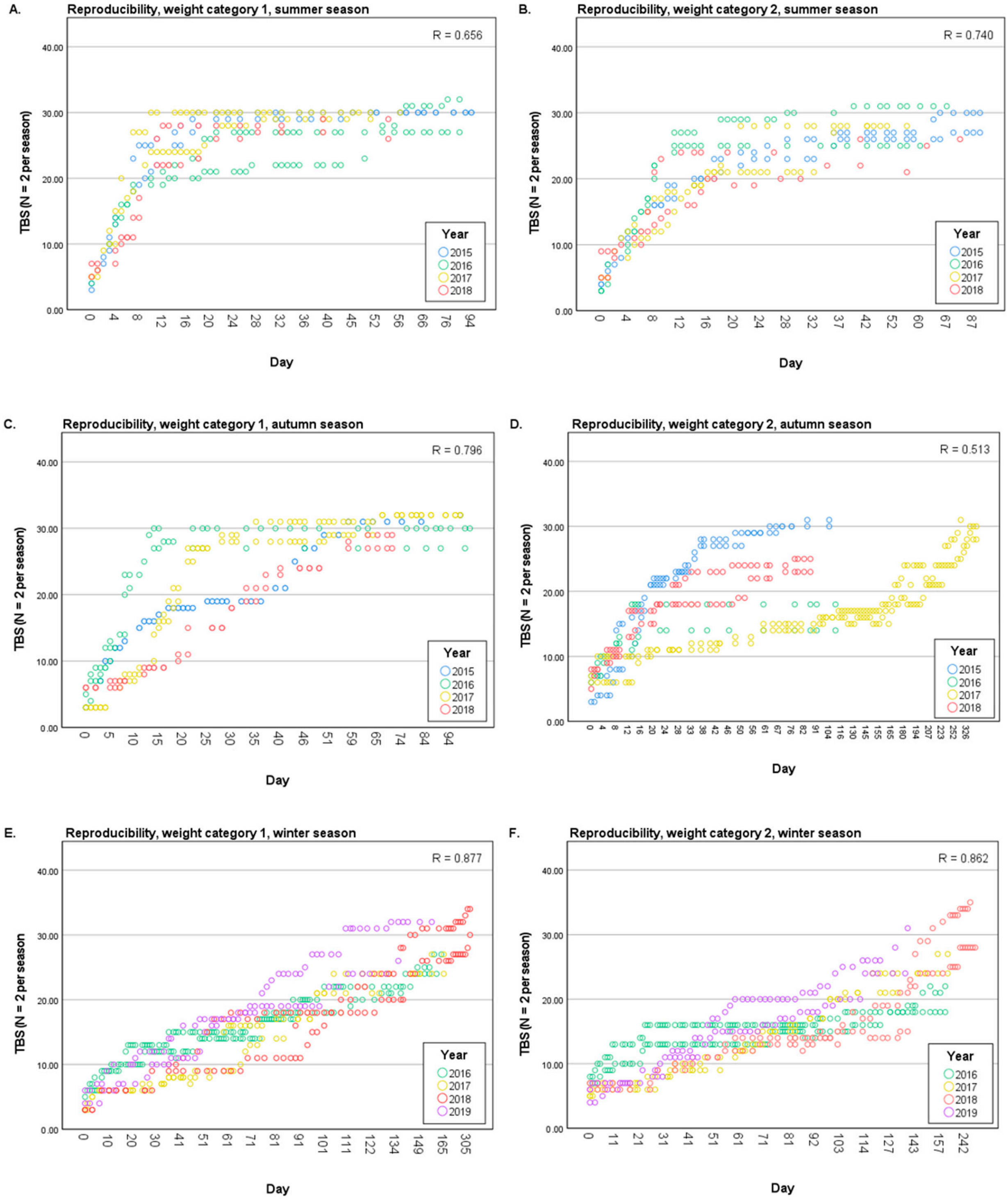

Figure S2. Reproducibility. Total Body Score of the pig cadavers against the number of days post-mortem, where all research yeas are shown (n = 2 per season), to show the progress in decomposition. A: Summer season weight cat. 1. B: Summer season weight cat. 2. C: Autumn season weight cat. 1. D: Autumn season weight cat. 2. E: Winter season weight cat. 1. F: Winter season weight cat. 2

Table S2. Results of the linear regression on data including plateau phase. The correlation, the Residual Sum of Squares and the slope can be found in the table, as well as the average reached maximum TBS. The asterisks indicates that the decomposition rate of this weight category was higher compared to the other weight category in the same season

Research years	Season and weight category	Average reached maximum TBS	Correlation	Residual Sum of Squares	Slope
2015/2016	Summer, cat. 1	30	0.649	2394.020	0.193
	Autumn, cat. 1	32	0.932	549.788	0.285
	Winter, cat. 1*	25	0.958	325.135	0.109
	Spring, cat. 1*	30	0.932	1030.382	0.330
	Summer, cat. 2*	29	0.811	1135.136	0.216
	Autumn, cat. 2*	31	0.854	1730.808	0.302
	Winter, cat. 2	20	0.821	491.408	0.056
	Spring, cat. 2	23	0.963	295.831	0.231
2016/2017	Summer, cat. 1	30	0.788	954.778	0.204
	Autumn, cat. 1*	29	0.647	3330.002	0.185
	Winter, cat. 1*	26	0.962	353.874	0.155
	Spring, cat. 1*	27	0.801	1636.998	0.217
	Summer, cat. 2*	28	0.665	1832.563	0.269
	Autumn, cat. 2	16	0.591	411.860	0.070
	Winter, cat. 2	26	0.980	148.010	0.141
	Spring, cat. 2	21	0.882	562.111	0.177
2017/2018	Summer, cat. 1	30	0.699	1835.119	0.367
	Autumn, cat. 1*	32	0.807	3632.349	0.294
	Winter, cat. 1	32	0.893	1724.101	0.103
	Spring, cat. 1*	29	0.918	663.638	0.320
	Summer, cat. 2*	28	0.882	640.498	0.449
	Autumn, cat. 2	29	0.953	530.795	0.060
	Winter, cat. 2*	32	0.905	1336.840	0.104
	Spring, cat. 2	25	0.937	416.363	0.265
2018/2019	Summer, cat. 1*	28	0.755	1093.882	0.454
	Autumn, cat. 1*	28	0.975	182.601	0.367
	Winter, cat. 1*	29	0.957	531.860	0.187
	Spring, cat. 1*	27	0.726	2377.807	0.255
	Summer, cat. 2	26	0.703	716.054	0.246
	Autumn, cat. 2	24	0.863	570.465	0.190
	Winter, cat. 2	28	0.953	351.139	0.166
	Spring, cat. 2	23	0.924	631.917	0.253
All years together	Summer, cat. 1	29	0.656	7745,968	0,225
	Autumn, cat. 1*	30	0.796	9443,602	0,271
	Winter, cat. 1*	28	0.877	5750,624	0,115
	Spring, cat. 1*	28	0.840	6476,971	0,278
	Summer, cat. 2*	28	0.740	5816,077	0,264
	Autumn, cat. 2	25	0.513	12270,024	0,042
	Winter, cat. 2	26	0.862	4391,745	0,097
	Spring, cat. 2	23	0.911	2504,902	0.228

Table S3. Results of the linear regression on data excluding plateau phase. Here it was decided to cut the TBS as soon as a cadaver reached a plateau phase in the advanced decay phase. The correlation, the Residual Sum of Squares and the slope can be found in the table, as well as the average reached maximum TBS excluding the plateau phase and the plateau phase cut-off point. The asterisks indicates that the decomposition rate of this weight category was higher compared to the other weight category in the same season

Research years	Season and weight category	Average maximum reached TBS excluding plateau phase	Cut-off point TBS (N=2 per season)	Correlation	Residual Sum of Squares	Slope
2015/2016	Summer, cat. 1*	30	30 and 30	0.777	1186.860	0.543
	Autumn, cat. 1	32	-	0.932	549.788	0.285
	Winter, cat. 1*	25	-	0.958	325.135	0.109
	Spring, cat. 1*	30	-	0.932	1030.382	0.330
	Summer, cat. 2	29	27 and 30	0.843	762.536	0.272
	Autumn, cat. 2*	31	-	0.854	1730.808	0.302
	Winter, cat. 2	20	-	0.821	491.408	0.056
	Spring, cat. 2	23	-	0.963	295.831	0.231
2016/2017	Summer, cat. 1	30	-	0.788	954.778	0.204
	Autumn, cat. 1*	29	30 and 27	0.649	2585.598	0.251
	Winter, cat. 1*	26	-	0.962	353.874	0.155
	Spring, cat. 1*	27	-	0.801	1636.998	0.217
	Summer, cat. 2*	28	31 and 25	0.851	710.891	0.740
	Autumn, cat. 2	16	Not in advanced decay	0.591	411.860	0.070
	Winter, cat. 2	26	-	0.980	148.010	0.141
	Spring, cat. 2	21	-	0.882	562.111	0.177
2017/2018	Summer, cat. 1*	30	30 and 29	0.800	747.527	0.799
	Autumn, cat. 1*	32	32 and 32	0.865	2197.133	0.453
	Winter, cat. 1	32	-	0.893	1724.101	0.103
	Spring, cat. 1*	29	29 and 29	0.938	375.611	0.410
	Summer, cat. 2	28	28 and 28	0.881	361.492	0.544
	Autumn, cat. 2	29	-	0.953	530.795	0.060
	Winter, cat. 2*	32	-	0.905	1336.840	0.104
	Spring, cat. 2	25	25 and 24	0.952	257.056	0.314
2018/2019	Summer, cat. 1*	28	-	0.755	1093.882	0.454
	Autumn, cat. 1*	28	27 and 29	0.983	88.825	0.429
	Winter, cat. 1*	29	-	0.957	531.860	0.187
	Spring, cat. 1	27	25 and 28	0.818	779.431	0.381
	Summer, cat. 2	26	-	0.703	716.054	0.246
	Autumn, cat. 2	24	25 and 23	0.874	458.687	0.227
	Winter, cat. 2	28	-	0.953	351.139	0.166
	Spring, cat. 2*	23	24 and 22	0.964	214.416	0.348
All years together	Summer, cat. 1	29	See TBS above	0.669	5743.825	0.261
	Autumn, cat. 1*	30	See TBS above	0.816	7088.116	0.332
	Winter, cat. 1*	28	-	0.877	5750,624	0.115
	Spring, cat. 1*	28	See TBS above	0.858	4956.773	0.294
	Summer, cat. 2*	28	See TBS above	0.747	4068.596	0.316
	Autumn, cat. 2	25	See TBS above	0.516	11992.685	0.042
	Winter, cat. 2	26	-	0.862	4391,745	0.097
	Spring, cat. 2	23	See TBS above	0.908	2263.879	0.240

Bibliography

1. Campobasso, C. P., Di Vella, G., & Introna, F., 2001. Factors affecting decomposition and Diptera colonization. For Sci Int *120*(1-2), 18-27. DOI: https://doi.org/10.1016/s0379-0738(01)00411-x

2. Mann, R. W., Bass, W. M., & Meadows, L. (1990) Time since death and decomposition of the human body: variables and observations in case and experimental field studies. J of Forensic Sci 35(1):103-111. DOI: https://doi.org/10.1520/JFS12806J

3. Alfsdotter, C., & Petaros, A. (2021) Outdoor human decomposition in Sweden: A retrospective quantitative study of forensic-taphonomic changes and postmortem interval in terrestrial and aquatic settings. J of Forensic Sci, 66(4):1348-1363. DOI: https://doi.org/10.1111/1556-4029.14719

4. Meyer, J., Anderson, B., & Carter, D. O. (2013) Seasonal variation of carcass decomposition and gravesoil chemistry in a cold (Dfa) climate. J of Forensic Sci 58(5):1175-1182. DOI: https://doi.org/10.1111/1556-4029.12169

5. Roberts, L. G., & Dabbs, G. R. A. (2015) Taphonomic Study Exploring the Differences in Decomposition Rate and Manner between Frozen and Never Frozen Domestic Pigs (Sus scrofa). J of Forensic Sci 60(3):588-594. DOI: https://doi.org/10.1111/1556-4029.12717

6. Giles, S. B., Harrison, K., Errickson, D., & Márquez-Grant, N. (2020) The effect of seasonality on the application of accumulated degree-days to estimate the early post-mortem interval. Forensic Sci Int 315:110419. DOI: https://doi.org/10.1016/j.forsciint.2020.110419

7. Dabbs, G. R., Connor, M., & Bytheway, J. A. (2016) Interobserver reliability of the total body score system for quantifying human decomposition. J of Forensic Sci 61(2):445-451. DOI: https://doi.org/10.1111/1556-4029.12962

8. Forbes, M. N., Finaughty, D. A., Miles, K. L., & Gibbon, V. E. (2019) Inaccuracy of accumulated degree day models for estimating terrestrial post-mortem intervals in Cape Town, South Africa. Forensic Sci Int 296:67-73. DOI: https://doi.org/10.1016/j.forsciint.2019.01.008

9. Dautartas, A., Kenyhercz, M. W., Vidoli, G. M., Meadows Jantz, L., Mundorff, A., & Steadman, D. W. (2018) Differential decompo-sition among pig, rabbit, and human remains. J of Forensic Sci 63(6):1673-1683. DOI: https://doi.org/10.1111/1556-4029.13784

10. Keough, N., Myburgh, J., & Steyn, M. (2017) Scoring of decomposition: a proposed amendment to the method when using a pig model for human studies. J of Forensic Sci 62(4):986-993. DOI: https://doi.org/10.1111/1556-4029.13390

11. Zhang, C., & Plastow, G. (2011) Genomic Diversity in Pig (Sus scrofa) and its Comparison with Human and other Livestock. Current genomics 12(2):138-146. DOI: https://doi.org/10.2174/138920211795564386

12. Duijst, W., Reijnders, U., Reijnen, G., & Dijkhuizen L. (2021) Ontbinding en het vaststellen van het postmortaal interval. In Handboek Forensische Geneeskunde; Gompel & Svacina, W. Duijst, U. Reijnders, G. Reijnen, & L. Dijkhuizen; Gompel & Svacina bv, ’s-Hertogenbosch, 282-311.

13. Madea, B., Henssge, C., Reibe, S., Tsokos, M., & Kernbach-Wighton, G. (2014) Postmortem changes and time since death. In Handbook of forensic medicine 75-133.

14. Meyer, M. R., & Maurer, H. H. (2014) Toxicokinetics and Toxicogenetics. In Handbook of forensic medicine; B. Madea; J. Wiley & Sons, Ltd, West Sussex, 889 – 899.

15. Saukko, P., & Knight, B. (2015) The Pathophysiology of Death. In Knight's forensic pathology. Taylor & Francis Group, LLC; CRC press: Bocan Raton, 55-94.

16. Zhou, C., & Byard, R. W. (2011) Factors and processes causing accelerated decomposition in human cadavers–an overview. J of Forensic and Leg Med 18(1):6-9. DOI: https://doi.org/10.1016/j.jflm.2010.10.003

17. Cordeiro, C., Ordóñez-Mayán, L., Lendoiro, E., Febrero-Bande, M., Vieira, D. N., & Muñoz-Barús, J. I. (2019) A reliable method for estimating the postmortem interval from the biochemistry of the vitreous humor, temperature and body weight. Forensic Sci Int 295:157-168. DOI: https://doi.org/10.1016/j.forsciint.2018.12.007

18. Miles, K. L., Finaughty, D. A., & Gibbon, V. E. (2020) A review of experimental design in forensic taphonomy: moving towards forensic realism. Forensic Sci Research 5(4):249-259. DOI: https://doi.org/10.1080/20961790.2020.1792631

19. Sutherland, A., Myburgh, J., Steyn, M., & Becker, P. J. (2013) The effect of body size on the rate of decomposition in a temperate region of South Africa. Forensic Sci Int 231(1-3):257-262. DOI: https://doi.org/10.1016/j.forsciint.2013.05.035

20. Matuszewski, S., Konwerski, S., Frątczak, K., & Szafałowicz, M. (2014) Effect of body mass and clothing on decomposition of pig carcasses. Int J of Leg Med 128(6):1039-1048. DOI: https://doi.org/10.1007/s00414-014-0965-5

21. Salimi, M., Chatrabgoun, O., Akbarzadeh, K., Oshaghi, M., Falahati, M. H., Rafizadeh, S., ... & Rassi, Y. (2018) Evaluation of insect succession patterns and carcass weight loss for the estimation of postmortem interval. J of Med Entomology 55(6):1410-1422. DOI: https://doi.org/10.1093/jme/tjy095

22. Wilson, A., Neilsen, P., Berry, R., Seckiner, D., & Mallett, X. (2020) Quantifying human post-mortem movement resultant from de-composition processes. Forensic Sci Int: Synergy 2:248-261. DOI: https://doi.org/10.1016/j.fsisyn.2020.07.003

23. Galloway, A. (1997) The process of decomposition: a model from the Arizona-Sonoran desert. In Forensic taphonomy: the postmortem fate of human remains; Taylor & Francis Group, LLC; CRC Press, Boca Raton, 139-150.

24. Lyu, Z., Wan, L. H., Yang, Y. Q., Tang, R., & Xu, L. Z. (2016) A checklist of beetles (Insecta, Coleoptera) on pig carcasses in the sub-urban area of southwestern China: A preliminary study and its forensic relevance. J of Forensic and Leg Med 41:42-48. DOI: https://doi.org/10.1016/j.jflm.2016.04.009

25. Tembe, D., & Mukaratirwa, S. (2021) Insect succession and decomposition pattern on pig carrion during warm and cold seasons in KwaZulu-Natal Province of South Africa. J of Medical Entomology 58(6):2047-2057. DOI: https://doi.org/10.1093/jme/tjab099

26. Reibe, S. (2015) Forensic Entomology. In Estimation of the time since death; B. Madea; Taylor & Francis Group, CRC Press Boca Raton, 249-258.

27. Cockle, D. L., & Bell, L. S. (2017) The environmental variables that impact human decomposition in terrestrially exposed contexts within Canada. Science & Justice 57(2):107-117. DOI: https://doi.org/10.1016/j.scijus.2016.11.001

28. Archer, M. S. (2004) Rainfall and temperature effects on the decomposition rate of exposed neonatal remains. Science & justice: J of the Forensic Sci Society 44(1):35-41. DOI: https://doi.org/10.1016/S1355-0306(04)71683-4

29. Galloway, A., Birkby, W., Jones, A., Henry, T., and Parks, B., 1989. Decay Rates of Human Remains in an Arid Environment. J of Forensic Sci 34(3), 607-16. DOI: https://doi.org/10.1520/JFS12680J

30. Matuszewski, S., Bajerlein, D., Konwerski, S., & Szpila, K. (2010). Insect succession and carrion decomposition in selected forests of Central Europe. Part 1: Pattern and rate of decomposition. Forensic Sci Int, *194*(1-3), 85-93. DOI: https://doi.org/10.1016/j.forsciint.2009.10.016

31. Díaz-Aranda, L. M., Martín-Vega, D., Gómez-Gómez, A., Cifrián, B., & Baz, A. (2018). Annual variation in decomposition and insect succession at a periurban area of central Iberian Peninsula. J of Forensic and Leg Med *56*, 21-31. DOI: https://doi.org/10.1016/j.jflm.2018.03.005

32. Goff, M. L., 2009. Early post-mortem changes and stages of decomposition in exposed cadavers. *Experimental and applied acarology*, *49*(1), 21-36. DOI: https://doi.org/10.1007/s10493-009-9284-9

33. Šuláková, H., & Barták, M., 2013. Forensically important Calliphoridae (Diptera) associated with animal and human decomposition in the Czech Republic: preliminary results. *Acta Musei Silesiae, Scientiae Naturales*, *62*(3), 255-266. DOI: https://doi.org/10.2478/cszma-2013-0024

34. Paczkowski, S., Nicke, S., Ziegenhagen, H., & Schütz, S., 2015. Volatile Emission of Decomposing Pig Carcasses (Sus scrofa domesticus L.) as an Indicator for the Postmortem Interval. J of Forensic Sci *60*, S130-S137. DOI: https://doi.org/10.1111/1556-4029.12638
